# The proton-activated G protein-coupled receptor GPR4 regulates the development of osteoarthritis via modulating CXCL12/CXCR7 signaling

**DOI:** 10.1038/s41419-021-04455-4

**Published:** 2022-02-14

**Authors:** Rong Li, Zijing Guan, Shuyan Bi, Fanhua Wang, Liang He, Xin Niu, Yu You, Yuwei Liu, Yi Ding, Stefan Siwko, Ning Wang, Ziming Zhang, Yunyun Jin, Jian Luo

**Affiliations:** 1 Shanghai Key Laboratory of Regulatory Biology, Institute of Biomedical Sciences and School of Life Sciences, East China Normal University, Shanghai, 200241 PR China; 2 Yangzhi Rehabilitation Hospital (Shanghai Sunshine Rehabilitation Center), Tongji University School of Medicine, Shanghai, 201619 PR China; 3 Department of Translational Medical Sciences, Institute of Biosciences and Technology, Texas A&M University Health Science Center, Houston, TX 77030 USA; 4 Department of Oncology and Metabolism, The University of Sheffield, Sheffield, UK; 5 Department of Pediatric Orthopedics, Xinhua Hospital, School of Medicine, Shanghai Jiaotong University, Shanghai, 200092 PR China

**Keywords:** Cartilage development, Osteoarthritis

## Abstract

Inflammatory diseases decrease the extracellular environmental pH. However, whether proton-activated G protein-coupled receptors (GPCRs) can regulate the development of osteoarthritis (OA) is largely unknown. In this study, we report that proton-activated GPR4 is essential for OA development. We found a marked increase in expression of the proton-activated GPR4 in human and mouse OA cartilage. Lentivirus-mediated overexpression of GPR4 in mouse joints accelerated the development of OA, including promotion of articular cartilage damage, synovial hyperplasia, and osteophyte formation, while Gpr4 knockout effectively attenuated the development of posttraumatic and aging-associated OA in mice. We also found that inhibition of GPR4 with the antagonist NE52-QQ57 ameliorated OA progression in mice, promoted extracellular matrix (ECM) production, and protected cartilage from degradation in human articular cartilage explants. Moreover, GPR4 overexpression upregulated matrix-degrading enzymes’ expression and inflammation factors under pro-inflammatory and slightly acidic conditions. Mechanistically, GPR4 suppressed chondrocyte differentiation and upregulated cartilage homeostasis through NF-κB/MAPK signaling activation by regulating CXCR7/CXCL12 expression. Together, our results take the lead to illustrate that proton-activated GPCR acts as a key regulator for OA pathogenesis in vivo, and support that GPR4 could be a promising therapeutic target for OA treatment.

## Introduction

Osteoarthritis (OA) is the most common degenerative disease of the joints and is characterized by cartilage degradation, synovial inflammation, subchondral bone sclerosis and osteophyte formation [1]. Over 300 million people globally are afflicted with OA, ultimately resulting in pain and physical disability [2]. Although increasingly studies have identified etiologic risk factors involved in the development of OA, including aging, mechanical, metabolic, or genetic factors, there are no effective clinical drugs that can prevent or treat the degeneration of articular cartilage associated with OA [3, 4].

The hallmark of OA is cartilage matrix breakdown, which is caused by excessive matrix-degrading enzyme activity and/or downregulation of chondrocyte extracellular matrix (ECM) molecules [5]. Among the matrix-degrading enzymes, MMPs and ADAMTS family proteases [6, 7], which degrade type II collagen and aggrecan, are known to play an important role in OA cartilage destruction. In addition, proinflammatory cytokines such as interleukin-1β (IL-1β), tumor necrosis factor-alpha (TNF-α), and interleukin-6 (IL-6) also contribute to the pathogenesis of OA. These proinflammatory cytokines can lead to upregulation of cartilage matrix-degrading enzymes (e.g., MMP13 and MMP3) [8, 9] and induction of nitric oxide synthase (iNOS) [10]. However, the regulatory mechanism of OA-related catabolic genes in chondrocytes remains unclear.

Extensive studies have indicated that inflammatory diseases decrease the pH of the extracellular environment [11, 12]. Increasing evidence shows that an acidic extracellular environment plays an important role in various pathological processes including bone remodeling and arthritis [13, 14]. However, there are controversial reports regarding environmental pH regulation in OA joints. It has been reported that the pH value of OA synovial fluid is 7.38–7.55 [15–17]. However, another study showed that the degenerating superficial zone of articular cartilage is acidic, in which a pH of 6.7–7.5 was observed in grade 0 cartilage surfaces, while the pH was 4.5–6.5 in grade 3 cartilage surfaces [18]. Acid sensing is essential for maintaining normal cell function through acid-sensing ion channels or proton-activated G protein-coupled receptors (GPCRs) [19, 20]. The proton-activated GPCR family includes 4 members, GPR4, GPR65 (TDAG8), GPR132 (G2A), and GPR68 (OGR1) [19]. GPR4, cloned as an orphan receptor in 1995 [21], is widely distributed and expressed in many human tissues, including lung, kidney, liver, dorsal raphe neurons, and heart [21, 22]. GPR4 is a novel proton-activated receptor, activated at a slightly acidic pH [19]. Previous studies have shown that in endothelial cells, GPR4 can sense acidic pH and induce the expression of a series of inflammatory factors including interleukins (ILs), as well as adhesion molecules including *VCAM1, ICAM1* [23]. The expression of RANKL in osteoblasts in an acidic environment is mediated by cAMP/PKA signals downstream of GPR4 activation [24]. In addition, GPR4 promotes VEGFA secretion via the p38 signaling pathway in squamous cell carcinoma of the head and neck [25]. In endothelial cells, GPR4 can active acidosis-induced ER stress and inflammation [26]. Recently, the anti-inflammatory role of the GPR4 antagonist NE52-QQ57 in rat antigen-induced rheumatoid arthritis as well as in a hyperalgesia and angiogenesis model has been reported [27]. An in vitro study indicates that a GPR4 antagonist inhibited the advanced glycation end product (AGE)-induced increased expression of several key inflammatory cytokines and signaling molecules in the human chondrocyte SW1353 cell line [28]. However, the functions of proton-activated GPCRs in regulating OA pathogenesis have not been studied.

In this study, we found that the expression of GPR4 was upregulated in human and mouse OA articular cartilage and overexpression of GPR4 or loss of GPR4 notably regulated articular cartilage degeneration, synovitis, osteophyte formation, and OA-associated pain. We identify activation of the CXCL12/CXCR7-NF-κB/MEK signaling pathway as critical for mediating GPR4 modulation of OA cartilage homeostasis. Our results support that the proton-activated GPCR GPR4 is an effective therapeutic target for OA treatment.

## Materials and methods

### Human OA cartilage samples

Human cartilage samples were obtained from individuals undergoing total knee arthroplasty, with the approval of the Human Ethics Committee of Shanghai Sixth People’s Hospital (2021-048). Basic clinical information of these patients is presented in Supplementary Table 2. Human cartilage explants were cultured as recently described [29]. Briefly, cartilage explants were cut into pieces of about 2 × 5 mm. They were then cultured in DMEM containing 10% FBS, supplemented with NE52-QQ57 (MedChemExpress, HY-1D1784, 10 μM) or vehicle for 1 week. Cartilage explants were then collected for histological analysis and mRNA expression level examination, while supernatant was collected for testing using the 1, 9, dimethyl methylene blue (DMMB) (Sigma-Aldrich, 341088-1G) assay.

### Mice

C57BL/6J male mice and *Gpr4*^−/−^ male mice were used for experimental OA studies. Ten- to twelve-week-old C57BL/6J (WT) male mice were purchased from GemPharmatech Co., Ltd. (Nanjing, China). Gpr4 global knockout mice (*Gpr4*^−/−^) were generated by the Animal Center of East China Normal University, using the CRISPR/Cas9 system in the C57BL/6J mouse strain. *Gpr4*^−/−^ mice were identified via polymerase chain reaction (PCR) using primers (sense, 5′GACAACAGCACGGGCACA3′; anti-sense, 5′AGCACAATGGCGATGAGG3′). All mice were maintained in pathogen-free barrier facilities and housed at 5 per cage with 12 h light/dark cycles. All animal experiments were performed in accordance with the procedures approved by the Animal Center of East China Normal University and the Animal Care and Ethics Committee of East China Normal University.

### Posttraumatic and aging-associated OA mouse models

Posttraumatic experimental OA was induced by DMM surgery in 10–12-week-old male mice as previously described [30]. Animals with the same genotype were randomly assigned to the sham and surgery groups. WT littermates were used as controls for *Gpr4*^−/−^ mice. The knee joints were harvested 8 weeks after DMM surgery for histological analyses. Experimental OA was also induced and mice were intra-articularly injected with lentivirus expressing GPR4 (Lenti-Gpr4 or empty vector lentivirus as negative control (Lenti-Ctrl). Lentivirus (1 × 10^9^ pfu in 10 μl) was injected into the intra-articular joint cavity using a microsyringe once per week for 7 weeks. Eight weeks after DMM surgery, the mice were sacrificed for micro-CT and histological analyses. The GPR4 antagonist NE52-QQ57 (MedChemExpress, HY-1D1784, 30 μM in 10 μl) was delivered into mouse knee joints via IA injection two times per week beginning 4 days after DMM surgery, and then 6 weeks after DMM surgery, the mice were sacrificed for micro-CT and histological analyses. *Gpr4*^−/−^ mice and WT littermates were maintained for 12 months as an aging-associated OA model.

### Micro-CT analysis of knee joints

The osteophyte development of mouse knee joints was detected by micro-CT analysis. The knee joints were scanned using X-ray microtomography (SKYSCAN 1272, Bruker micro-CT) under the conditions of 60 kV, 166.0 μA, and a resolution of 7 μm per pixel. Three-dimensional reconstruction of the knee joints was produced using CTVOL (Bruker micro-CT).

### Histology, immunohistochemistry, and immunofluorescence

Human cartilage samples were decalcified in 0.5 M EDTA at 4 °C for 7 days after being fixed in 4% paraformaldehyde (PFA), then embedded in paraffin and sectioned continuously (6 μm in thickness). The paraffin sections were stained with Safranin-O/Fast green. Human OA cartilage degradation was scored using the International Cartilage Repair Society (ICRS) grading system as described previously [31]. For immunohistochemistry staining, the sections were pre-treated using proteinase K mediated Antigen retrieval, which was performed with 20 μg/ml proteinase K for 20 min at 37 °C, and then sequentially treated with 3% H_2_O_2_, 0.1% Triton X-100 and blocking with 5% BSA for 1 h at room temperature. Next, sections were treated with GPR4 (1:100; Thermo Fisher; Waltham, MA, PA5-33710), MMP13 (1:100; Abcam; Cambridgeshire, UK, ab39012), or MMP3 (1:100; Proteintech; 17873-1-AP) antibodies overnight at 4 °C. Horseradish peroxidase (HRP)-conjugated secondary antibody was incubated for 1 h at room temperature and followed by treatment with ABC Kit reagents (Vector Laboratories, Burlingame, CA, USA). IHC signals were detected with the DAB Substrate Kit (Vector Laboratories, Burlingame, CA, USA). For fluorescence immunostaining, sections were incubated with Col2 (1:100; Abcam; Cambridgeshire, UK, ab34712) overnight at 4 °C and then incubated with secondary antibody conjugated with Alexa Fluor 488 (1:200; Invitrogen, Carlsbad, CA, USA) for 1 h at room temperature, while nuclei were counterstained with DAPI (Beyotime Institute of Biotechnology, Jiangsu, China). Images were acquired with an Olympus microsystems microscope (Olympus Corporation, Tokyo, Japan) and analyzed using ImageJ software (V1.52v).

For experiments using mouse models, the knee joints of mice were fixed in 4% PFA at 4 °C for 48 h, decalcified in 0.5 M EDTA for 14 days, and embedded in paraffin. Paraffin blocks were cut into sections with a thickness of 6 μm. The paraffin sections were stained with Safranin-O/Fast green and Hematoxylin & Eosin (H&E). The OARSI grading system was used to evaluate cartilage damage by blinded observers [32]. Synovitis was scored as previously described [33]. Osteophyte maturity and formation were quantified as previously described [34]. For immunohistochemistry staining, similarly to human cartilage, staining for Col X (1:1000; Abcam; Cambridgeshire, UK, ab58632), MMP13 (1:100; Abcam; Cambridgeshire, UK, ab39012), Col2 (1:100; Abcam; Cambridgeshire, UK, ab34712), GPR4 (1:100; Thermo Fisher; Waltham, MA, PA5-33710), and CXCR7 (1:200; Proteintech; 20423-1-AP) was performed. Images were acquired with an Olympus microsystems microscope (Olympus Corporation, Tokyo, Japan) and analyzed using ImageJ software (V1.52 v). Osteophyte size (0–3) and osteophyte maturity (0–3) were scored according to a previous study [34].

### OA pain assay

We assessed behavioral pain using the von Frey assay and the hotplate test. Mechano-sensitivity experiments were performed using von Frey filaments (Ugo Basile Biological Research Apparatus Company, Milan, Italy). For the thermal hyperalgesia test, mice were placed on the hot plate meter (Ugo Basile Biological Research Apparatus Company, Milan, Italy), setting PSU40. The mice were placed in a plexiglass cylinder and kept quiet for 1–2 h. The incubation period was recorded until licking and/or shaking or jumping of the hind paws, at which point the mice were immediately moved away from the hot plate. Each mouse was measured at 3 points at a time for 3 times, with an interval of 30 min for each time. Investigators were blinded to specific groups.

### Cell culture

Mouse primary chondrocytes from WT or *Gpr4*^*−/−*^ mice were isolated from articular cartilage postnatal day 4 pups and digested overnight with 0.1% collagenase. Primary chondrocytes were cultured in DMEM/F12 supplemented with 1% penicillin/streptomycin and 10% fetal bovine serum. Cells were passaged when confluence reached 80–90%. P1 cells were used for each experiment according to specific experimental requirements. The ATDC5 cell line was purchased from Riken Cell Bank (Ibaraki, Japan) and maintained in DMEM supplemented with 10% FBS and 1% penicillin/streptomycin.

### Mouse primary chondrocyte micromass culture and Alcian blue staining

For micromass culture, P1 chondrocytes (2.5 × 10^4^ cells in 12.5 μl) were dropped into round droplets in wells of a 24-well-plate, placed for attachment at 37 °C for 3–4 h, supplemented with 500 μl chondrocyte differentiation medium and cultured for 3 days, then subjected to Alcian blue staining and RNA extraction. For Alcian blue staining, micromass cultures were washed with PBS for 3 times, then fixed at room temperature for 15 min in 4% PFA, and stained with Alcian blue solution (1.0% Alcian blue in 0.1 N HCl) for 4 h. ddH_2_O washes were performed until no color was visible in the supernatant. Images were acquired with a Nikon camera and quantified using ImageJ software.

### Lentiviral construct packaging

Expression construct of mouse Gpr4 was subcloned into pLVX-IRES-ZsGreen1 vector by inserting the CDS of gene at Ecor I and Xba I sites. The GPR4 lentivirus was produced using a 3rd Generation Packaging system (including packing plasmids: PMD2G and PSPAX2; expression plasmid: PLVX-IRES-GPR4). Briefly, the plasmid was transfected with 293T cells using PEI as a normal transfection procedure. The supernatant was collected after 48 h, and the lentivirus particles were concentrated by ultracentrifugation at 50,000 × *g* (Beckman Coulter, USA) for 70 min at 4 °C. Then, the pellets were resuspended with 500 μl PBS and stored at −80 °C. The PFU of lentiviral particles were determined as previously reported [35].

### Reporter gene assay

Luciferase assay was performed in ATDC5 cells using an NF-κB Luciferase Reporter (Promega, USA). ATDC5 cells were plated at 25% confluence in 24-well tissue culture plates and NF-κB luciferase reporter (50 ng), GPR4 plasmid (500 ng), or vector (pcDNA3.1) were co-transfected with Renilla plasmid (10 ng) on the next day. Twenty-four hours after transfection, the cells were treated with IL-1β (1 ng/ml), with or without 10 μM NE52-QQ57 for 24 h. Then cells were harvested and luciferase activity was assessed with the Dual-Luciferase Reporter Assay System (Promega, USA).

### siRNA preparation, transfection, and quantitative real-time PCR

The sequence of the siRNA against CXCR7 was siRNA1: 5′GGAUGUGCACUUGUUUGACUAUGCA3′, siRNA2: 5′CACAGUAGCCGGAAGAUCAUCUUCU3′, obtained from Biotend (Shanghai, China). ATDC5 cells were plated in 24-well-plate at a density of 5 × 10^4^ cells/well. After 12–16 h, siRNA targeting CXCR7 (siCXCR7) (100 nM) was transfected using Lipofectamine 2000 (Thermo Fisher, 11668019) for 24 h and cells were treated with IL-1β (1 ng/ml) for 24 h. Total RNA was extracted using TRIzol reagent (MaGen, R4801-02). cDNA was synthesized using PrimeScript^TM^ RT reagent Kit (TaKaRa Bio, Kyoto, Japan). qRT-PCR was amplified with PCR using SYBR premixExTaq reagents (Yeasen, Shanghai, China) quantified by Step One Plus PCR system (AB Applied, Life Technologies, Thermo Fisher Scientific, Waltham, MA, USA). All qRT-PCR was performed in 3 replicates, and each target gene was normalized to glyceraldehyde-3-phosphate dehydrogenase (GAPDH). Relative gene expression levels were expressed as fold changes, using the 2^−ΔΔCT^ method. The primers used are summarized in Supplementary Table 3.

### Protein extraction and western blotting

ATDC5 cells were transfected with GPR4 or empty vector for 48 h using Lipofectamine 2000 (Invitrogen, Carlsbad, CA, USA). After 12 h of starvation, the cells were stimulated with 10 ng/ml IL-1β at 37 °C for 0, 5, 15, and 30 min, with or without the presence of NE52-QQ57 (10 μM). Total cell lysates were prepared in lysis buffer (150 mM NaCl, 50 mM Tris/HCl pH 7.5, 1% Triton X-100, 1 mM EDTA, 50 mM NaF, 10% (v/v) glycerol, protease inhibitors cocktail, and phosphatase inhibitors cocktail). The cell lysate was incubated on ice for 30 min and centrifuged at 12,000 rpm at 4 °C for 30 min. The protein concentration of the sample was determined by BCA assay (Thermo Fisher; 23225). The protein samples were then subjected to Western blot analysis using primary antibodies against MMP3 (1:1000; Proteintech, 17873-1-AP), MMP13 (1:1000; Servicebio, Wuhan, China), ERK (1:2000; Cell Signaling Technology, #4695), NF-κB p65 (1:1000; Cell Signaling Technology, #8242), p38 (1:1000; Cell Signaling Technology, #8690), p-SAPK/JNK (1:1000; Cell Signaling Technology, #9252), phospho-ERK (1:2000; Cell Signaling Technology, #4377), NF-κB p-p65 (1:1000; Cell Signaling Technology, #3033), phospho-38 (1:1000; Cell Signaling Technology, #9216), p-SAPK/JNK (1:1000; Cell Signaling Technology, #4668), and GAPDH (1:5000; Abcam). Densitometry of protein bands was quantified by using ImageJ software (V1.52 v). IκBα was normalized to GAPDH, p-P65, p-JNK, p-ERK, p-P38 were normalized to total p65, JNK, ERK, and p38, respectively.

### p65 nuclear localization by immunofluorescence

ATDC5 cells were transfected with GPR4 or empty vector for 48 h in 24-well-plate at a density of 6 × 10^4^ cells/well. The cells were stimulated with 1 ng/ml IL-1β at 37 °C for 12 h, with or without the presence of NE52-QQ57 (10 μM). The cells were fixed in 4% PFA for 10 min, permeabilized with 0.1% Triton X-100 in PBS for 10 min, and blocked with 5% BSA for 1 h at room temperature. The cells were then treated with anti-NF-κB p65 (1:200; Cell Signaling Technology) overnight at 4 °C and incubated with secondary antibody conjugated with Alexa Fluor 594 (1:200; Invitrogen, Carlsbad, CA, USA) for 1 h at room temperature. The nuclei were counterstained with DAPI (Beyotime Institute of Biotechnology, Jiangsu, China). Images were acquired with an Olympus microsystems microscope (Olympus Corporation, Tokyo, Japan) and analyzed using ImageJ software (V1.52v).

### Statistical analysis

All experiments were performed independently at least three times. Data are presented as mean ± s.d. For qRT-PCR, data are expressed as relative fold changes. The statistical significance of two independent groups was analyzed by the unpaired two-tailed Student’s *t* test, multiple comparisons were performed using one-way analysis of variance (ANOVA) followed by Bonferroni’s test, and two-way ANOVA followed by Tukey’s post hoc test using Prism 8 software (GraphPad). The level of significance was accepted at *p* < 0.05.

## Results

### GPR4 expression was upregulated in human and mouse OA articular cartilage

**Fig. 1:**
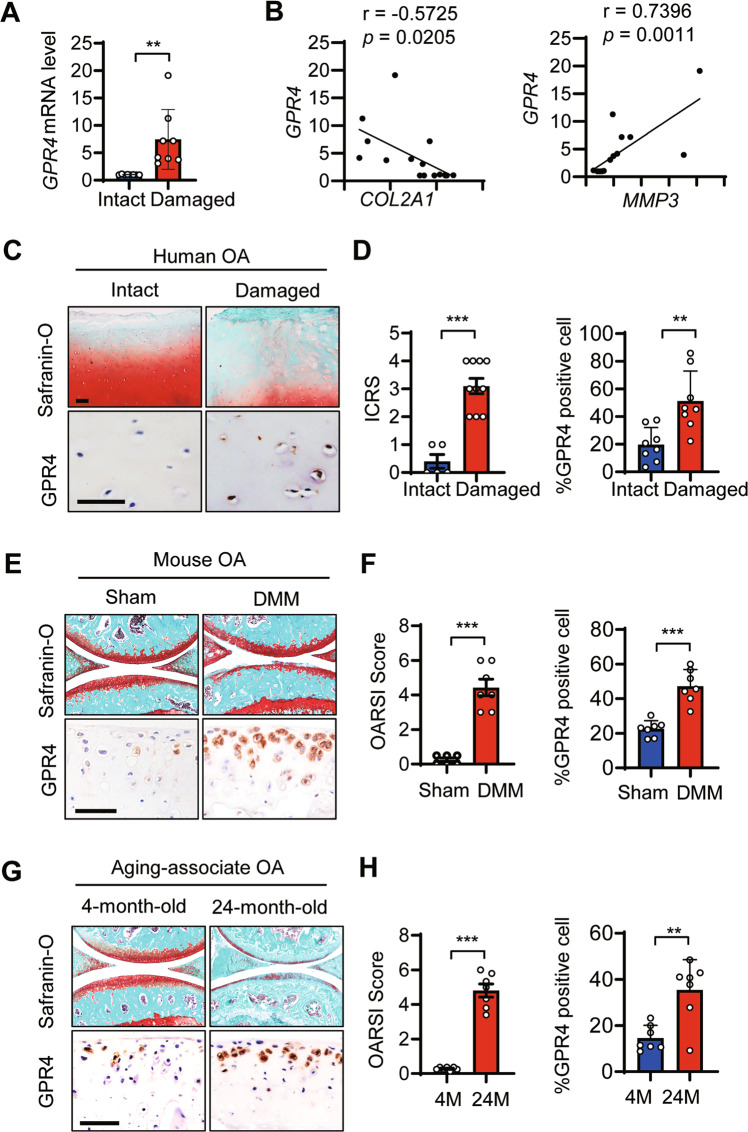
GPR4 expression was upregulated in human and mouse OA articular cartilage. **A** The mRNA levels of *GPR4* in intact and damaged regions of articular cartilage from OA patients (*n* = 8) were determined by qRT-PCR. Data are expressed as mean ± s.d. **p < 0.01 by Student’s two-tailed *t* test. **B** Correlation between the expression of *GPR4* and *COL2A1* (left), or *MMP3* (right) in intact and damaged regions of articular cartilage from OA patients (*n* = 8) by qRT-PCR. Pearson’s correlation analysis was performed. **C**, **D** Representative images of Safranin-O staining and immunohistochemistry (IHC) staining of GPR4 in intact and damaged regions of articular cartilage from human OA patients (**C**), Scale bar, 50 μm (*n* = 8 patients per group). International Cartilage Repair Society (ICRS) scores of human OA cartilage were analyzed (**D**, left) and GPR4 positive cells were quantified by IHC (**D**, right). *n* = 8. Data are expressed as mean ± s.d. **p < 0.01, ***p < 0.001 by Student’s two-tailed *t* test. **E**–**H** Staining of articular cartilage sections with Safranin-O and GPR4 IHC from sham-operated (*n* = 6) or DMM-operated (*n* = 7) mice (**E**) or 4- or 24-month-old mice (*n* = 7). Scale bars, 50 μm. **G** The corresponding OARSI (Osteoarthritis Research Society International) scores were assessed (**F** and **H**, left) and GPR4 expression was quantified (**F** and **H**, right), respectively. *n* = 7. Data are expressed as mean ± s.d. **p < 0.01, ***p < 0.001 by Student’s two-tailed *t* test.

To elucidate the potential role of the proton-activated GPCR GPR4 in OA pathogenesis, we collected human articular cartilage tissues from eight OA patients undergoing total knee replacement, and examined the expression levels of GPR4 in the OA cartilage tissues (Damaged) and relatively undamaged regions (Intact) of arthritic cartilage. Quantitative reverse transcription (qRT-PCR) analysis revealed that *GPR4* expression was markedly elevated in damaged regions compared with intact tissues in the OA patients. *GPR4* also showed a significant positive correlation with *MMP3*, a marker for ECM degradation, and a negative correlation with the *COL2A1*, a marker for ECM production (Fig. 1A, B). Consistent with our qRT-PCR results, the GPR4 protein level was increased in damaged OA cartilage but was barely detectable in intact regions using IHC staining (Fig. 1C, D). We further examined the expression level of GPR4 in the articular cartilage of both the destabilization of the medial meniscus (DMM) surgery-induced posttraumatic OA mouse model [30] and the aging-associated OA mouse model [36]. Similarly, the GPR4 protein level was notably increased in the cartilage of these two mouse OA models (Fig. 1E–H). All our results indicate that the expression of GPR4 was positively correlated with OA.

### Overexpression of GPR4 in joints promoted posttraumatic OA progression in vivo

**Fig. 2:**
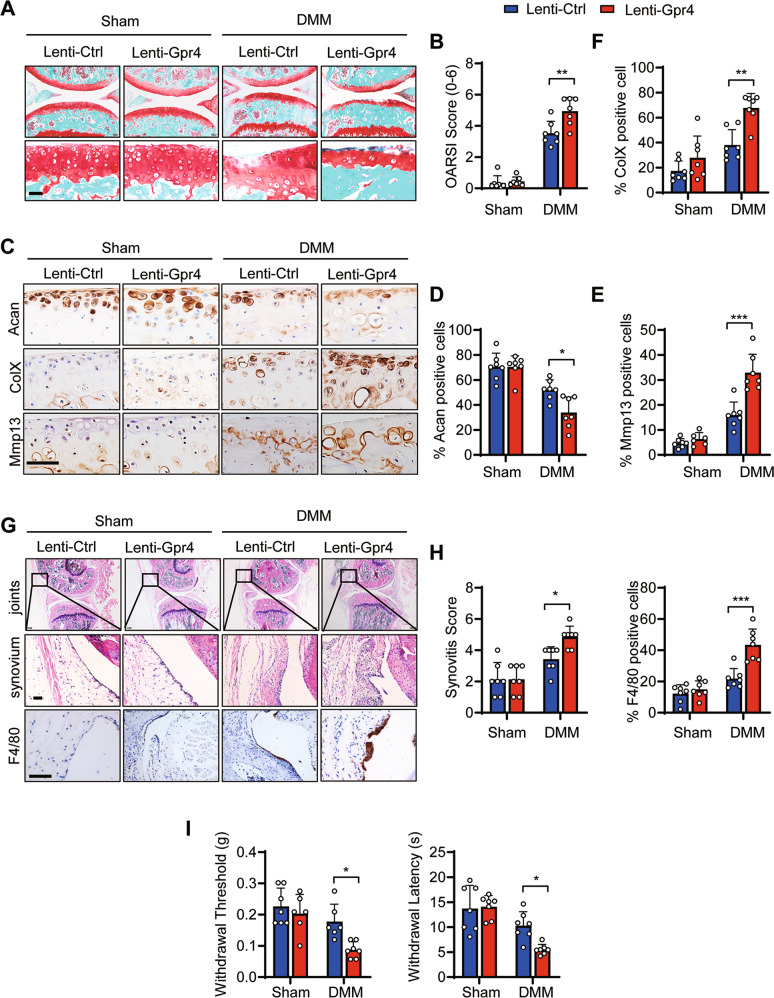
Lentivirus-mediated overexpression of GPR4 in mouse joint accelerated the development of posttraumatic OA. **A**, **B** Representative images of Safranin-O staining of mouse joints injected with lentivirus expressing GPR4 (Lenti-Gpr4) or empty control (Lenti-Ctrl). Four days after DMM surgery, the mice were IA injected with lentivirus once a week. Eight weeks later, the joints were collected and subject to Safranin-O staining (**A**). Scale bars, 50 μm. OARSI scores (**B**) were analyzed. Sham groups (*n* = 7 mice), DMM groups (*n* = 7 mice). Data are expressed as mean ± s.d. **p < 0.01 using two-way ANOVA followed by Tukey’s post hoc test. **C**–**F** Representative images of indicated antibody IHC staining of knee joint sections taken 8 weeks after DMM or Sham operation with Lenti-Gpr4 or Lenti-Ctrl injection. The corresponding quantitative analyses were shown in (**D**–**F**). Scale bars, 50 μm. *n* = 7 per group. Data are expressed as mean ± s.d. *p < 0.05, **p < 0.01, ***p < 0.001 using two-way ANOVA followed by Tukey’s post hoc test. **G**, **H** The joints were collected and subjected to H&E and IHC staining. Scale bars, 50 μm. **G** Synovitis scores were determined based on H&E staining (**H** left, *n* = 7) and F4/80 positive cells were quantified (**H** right, *n* = 7). Data are expressed as mean ± s.d. *p < 0.05, ***p < 0.001 using two-way ANOVA followed by Tukey’s post hoc test. **I** Von Frey assay (left) and thermal hyperalgesia test (right) were performed 8 weeks after DMM or Sham operation with Lenti-Gpr4 or Lenti-Ctrl injection. *n* ≥ 6. Data are expressed as mean ± s.d. *p < 0.05 using two-way ANOVA followed by Tukey’s post hoc test.

To investigate the role of GPR4 in the pathogenesis of OA, we employed the posttraumatic OA mouse model. Four days after DMM surgery, lentivirus expressing GPR4 (Lenti-Gpr4) or empty control (Lenti-Ctrl) virus were intra-articular (IA) injected (Supplementary Fig. 1A). IHC staining showed that IA injection of Lenti-Gpr4 triggered effective overexpression of GPR4 in cartilage and meniscus at 8 weeks after DMM surgery (Supplementary Fig. 1B). Compared with the control group, our results showed that GPR4 overexpression significantly aggravated DMM surgery-induced OA and cartilage destruction as assessed by Safranin-O staining (Fig. 2A, B). Moreover, the cartilage anabolic marker Aggrecan (Acan) was decreased (Fig. 2C, D) while the cartilage catabolic marker Mmp13 (Fig. 2C, E) and the chondrocyte hypertrophic marker ColX (Fig. 2C, F) were increased in GPR4 overexpression mice compared to control mice 8 weeks post DMM surgery. Our micro-CT results also showed that overexpression of GPR4 remarkably increased osteophyte formation including osteophyte size and osteophyte maturity in this mouse model (Supplementary Fig. 1C, D). Furthermore, GPR4 overexpression promoted DMM surgery-induced synovitis and macrophage infiltration by H&E staining and F4/80 immunostaining (Fig. 2G, H). As a result, GPR4 overexpression mice had lower paw-withdrawal thresholds and a shorter hotplate response time compared with the control mice after DMM surgery (Fig. 2I), indicating that overexpression of GPR4 enhanced OA pain. Together, all the results demonstrate that GPR4 is a positive regulator in OA pathogenesis.

### Genetic ablation of *Gpr4* prevented the development of posttraumatic and aging-associated OA

To determine the role of endogenous GPR4 in OA pathogenesis, we generated Gpr4 knockout (KO) mice using the CRISPR/Cas9 technology (Supplementary Fig. 2A, C). *Gpr4* knockout had little effect on cartilage development including the morphology and area of growth plates (Supplementary Fig. 2D,E).

**Fig. 3:**
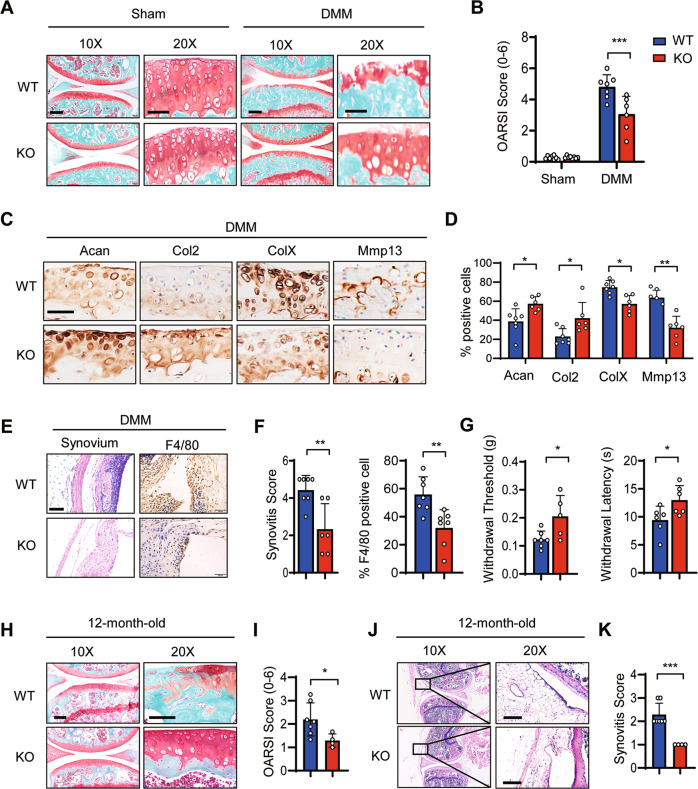
Gpr4 knockout blocked the development of posttraumatic and aging-associated OA in mice. **A**, **B** Representative images of Safranin-O staining of the knee joint at 8 weeks after sham operation (Sham) or DMM surgery (DMM) from WT mice (*n* = 7) and *Gpr4* knockout (KO) littermate mice (*n* = 6). The corresponding OARSI scores are shown (**B**). Scale bar, 50 μm. Data are expressed as mean ± s.d. ***p < 0.001 using two-way ANOVA followed by Tukey’s post hoc test. **C**, **D** Representative images of IHC staining for anabolic factors Acan and Col2, and catabolic factors Mmp13 and ColX from knee joint sections of Gpr4 WT and KO littermate mice 8 weeks after DMM surgery. Scale bars, 50 μm (**C**). Quantitative analysis is shown in (**D**). WT mice (*n* = 7) and *Gpr4* knockout (KO) littermate mice (*n* = 6). Data are expressed as mean ± s.d. *p < 0.05, **p < 0.01 by unpaired two-tailed Student’s *t* test. **E**, **F** Gpr4 knockout relieved synovial inflammation and macrophage infiltration in an OA mouse model. Eight weeks after DMM surgery, mouse joints were collected and subjected to H&E and F4/80 IHC staining (**E**). Synovitis scores were calculated based on the H&E staining (**F** left) and F4/80 positive cells were quantified (**F** right) in sections from WT (*n* = 7) and Gpr4 knockout (KO) littermate mice (*n* = 6). Data are expressed as mean ± s.d. **p < 0.01 by unpaired two-tailed Student’s *t* test. **G** Von Frey assay (left) and thermal hyperalgesia test (right) were performed 8 weeks after DMM surgery was performed on WT (*n* = 7) and Gpr4 knockout (KO) littermate mice (*n* = 6). Data are expressed as mean ± s.d. *p < 0.05 by unpaired two-tailed Student’s *t* test. **H**, **I** Representative images of Safranin-O staining of the knee joint from 12-month-old Gpr4 KO (*n* = 4) and wild type (WT) littermate mice (*n* = 7). The corresponding OARSI scores were evaluated (**I**). Scale bars, 100 μm. Data are presented as mean ± s.d. *p < 0.05 using unpaired two-tailed Student’s *t* test. **J**, **K** Gpr4 deletion relieved synovial inflammation in aging mice. The joints of 12-month-old mice were collected and subjected to H&E staining (**J**). Synovitis scores were determined based on the H&E staining (**K**) of sections from GPR4 KO mice (*n* = 4) and wild-type (WT) littermate mice (*n* = 7). Data are expressed as mean ± s.d. ***p < 0.001 by unpaired two-tailed Student’s *t* test. Scale bars, 100 μm.

We investigated the effect of GPR4 on the development of OA in posttraumatic and aging-associated OA mouse models. In the posttraumatic OA model, we found that knocking out *Gpr4* markedly relieved OA and cartilage degradation as examined through Safranin-O staining (Fig. 3A, B). The cartilage anabolic marker Acan and Col2 were increased while the cartilage catabolic marker Mmp13 and the chondrocyte hypertrophic marker ColX were decreased in the *Gpr4* deficient group compared to the WT group (Fig. 3C, D). Synovitis and macrophage infiltration were notably decreased in *Gpr4*^*−/−*^ mouse synovium compared to WT mice (Fig. 3E, F). Behavioral pain test results also showed that *Gpr4*^*−/−*^ mice had higher paw-withdrawal thresholds and longer hotplate response times compared with WT mice after DMM surgery (Fig. 3G). However, there were no significant differences in osteophyte size and osteophyte maturity between *Gpr4* knockout and WT mouse joints (Supplementary Fig. 3A). Similar results were obtained from the aging-associated OA mouse model that *Gpr4* knockout remarkably relieved OA and cartilage degradation as determined by Safranin-O staining (Fig. 3H, I), and suppressed synovitis by H&E staining of synovium (Fig. 3J, K). Together, our results demonstrated that depletion of GPR4 inhibited the development of posttraumatic and aging-associated OA.

### GPR4 antagonist treatment ameliorated OA progression

**Fig. 4:**
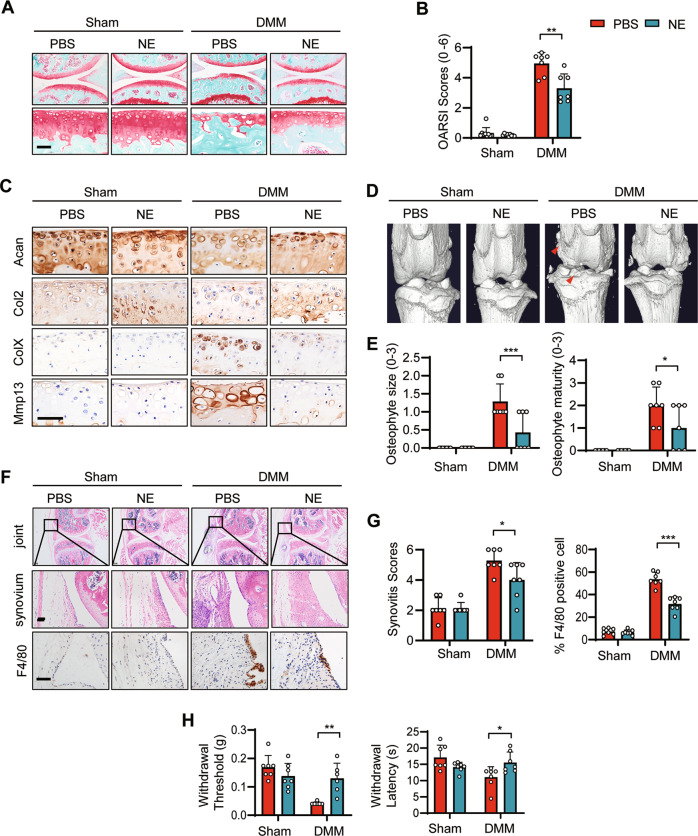
Pharmaceutical inhibition of GPR4 suppressed the progression of OA. **A**, **B** Representative images of Safranin-O staining of the knee joint at 6 weeks after DMM surgery. The mice were IA injected with 10 μl PBS or NE52-QQ57 (NE, 30 μM) two times per week. Six weeks later, the mouse joints were collected and subjected to staining (**A**). The corresponding OARSI scores were analyzed (**B**). *n* = 7 mice per group. Data are presented as mean ± s.d. **p < 0.01, using two-way ANOVA followed by Tukey’s post hoc test. Scale bar, 50 μm. **C** Representative images of IHC staining (for anabolic factors Acan and Col2, catabolic factors Mmp13 and ColX) of sections from PBS or GPR4 antagonist NE52-QQ57 (NE) IA injected mice (**C**). Scale bars, 50 μm. **D** Representative three-dimensional micro-CT images of the knee joints in sagittal view. The red arrows indicate osteophytes. **E** Osteophyte formation was scored by assessing osteophyte size (left) and maturity (right) from PBS or GPR4 antagonist NE52-QQ57 (NE) IA injected mice. Six weeks post DMM surgery, the mouse joints were collected and subjected to osteophyte score analysis. *n* = 7 mice per group. Data are expressed as mean ± s.d. *p < 0.05, ***p < 0.001 two-way ANOVA followed by Tukey’s post hoc test was used for statistical analysis. **F** Treatment of GPR4 antagonist NE52-QQ57 (NE) relieved synovial inflammation and microphage infiltration in an OA mouse model. Six weeks after DMM surgery, mouse joints were collected and subjected to H&E and F4/80 IHC staining. Scale bars, 50 μm. **G** Synovitis scores were evaluated based on H&E staining (**G** left, *n* = 7) and F4/80 positive cells were quantified (**G** right, *n* = 7). Data are expressed as mean ± s.d. *p < 0.05, ***p < 0.001 by two-way ANOVA followed by Tukey’s post hoc test. **H** Von Frey assay (left) and thermal hyperalgesia test (right) were performed 6 weeks after DMM surgery with PBS or NE52-QQ57 (NE) treatment (*n* = 7). Data are presented as mean ± s.d. *p < 0.05, using two-way ANOVA followed by Tukey’s post hoc test.

We next asked whether targeting GPR4 is effective for OA treatment. We evaluated the effect of the GPR4 antagonist NE52-QQ57 on a posttraumatic OA mouse model. NE52-QQ57 (NE) treatment notably inhibited the progression of OA and cartilage destruction, and markedly reduced OARSI scores after IA injection (Fig. 4A, B). Consistent with these results, NE treatment upregulated the cartilage anabolic markers Acan and Col2, while it reduced levels of the cartilage catabolic marker Mmp13 and the hypertrophic marker ColX (Fig. 4C). NE injection also inhibited DMM surgery-induced osteophyte formation (Fig. 4D, E) and synovitis including macrophage infiltration (Fig. 4F, G). As a result, NE treatment significantly reduced OA pain measured by the Von Frey assay and the thermal hyperalgesia test (Fig. 4H).

### GPR4 antagonist promoted ECM production and protected cartilage from degradation in human articular cartilage explants

**Fig. 5:**
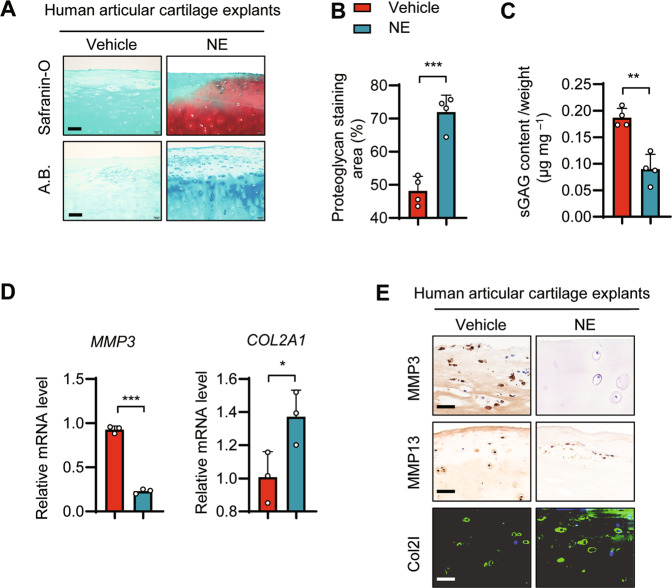
Inhibition of GPR4 promoted ECM production and suppressed cartilage catabolism in human articular cartilage explants. **A**–**C** Representative images of Safranin-O staining and Alcian blue (A.B.) staining in human articular cartilage explants. Relatively intact articular cartilage explants were cut from knee OA patients, and then the explants were treated with vehicle control or GPR4 antagonist NE52-QQ57 (NE, 10 μM) for 1 week. The explants were collected and subject to Safranin-O staining (**A**). Proteoglycan staining area (%) was analyzed (**B**). The sulfated GAG (sGAG) released from explants was measured by 1, 9, dimethyl methylene blue (DMMB) binding assay (**C**). The released amount of sGAG was normalized to the wet weight of the cartilage explant. *n* = 4, Data are presented as mean ± s.d. **p < 0.01, ***p < 0.001, using unpaired two-tailed Student’s *t* test. Scale bar, 100 μm. **D** The mRNA levels of *MMP3* and *COL2A1* in human articular cartilage explants treated with or without NE52-QQ57 (10 μM) for 1 week were determined by qRT-PCR. *n* = 3, data are presented as mean ± s.d. *p < 0.05, ***p < 0.001 using unpaired two-tailed Student’s *t* test. **E** Representative images of IHC and immunofluorescence staining of MMP13, MMP3, and COL2 of human articular cartilage explants treated with or without NE52-QQ57 (NE, 10 μM) for 1 week. Scale bar, 50 μm.

To assess the role of GPR4 in human cartilage, we isolated articular cartilage from relatively intact regions of arthritic cartilage during total knee arthroplasty, and treated the cartilage explants with the GPR4 antagonist NE for 1 week. NE strikingly promoted the proteoglycan content and increased the proteoglycan area observed in the Safranin-O and Alcian blue staining assay (Fig. 5A, B). NE treatment also inhibited the release of glycosaminoglycans (GAG, an ingredient of cartilage ECM), as examined using the 1, 9, dimethyl methylene blue (DMMB) assay (Fig. 5C). Furthermore, NE treatment enhanced the expression of *COL2A1* while inhibiting the expression of *MMP3* by qRT-PCR (Fig. 5D). Similar results were obtained from IHC staining where NE treatment promoted the expression of COL2 and suppressed the expression of MMP3 and MMP13 in human articular cartilage explants (Fig. 5E). All the data indicated that GPR4 could be a promising drug target for OA treatment.

### GPR4 upregulated catabolic marker gene expression under pro-inflammatory and slightly acidic conditions, suppressing chondrocyte differentiation

**Fig. 6:**
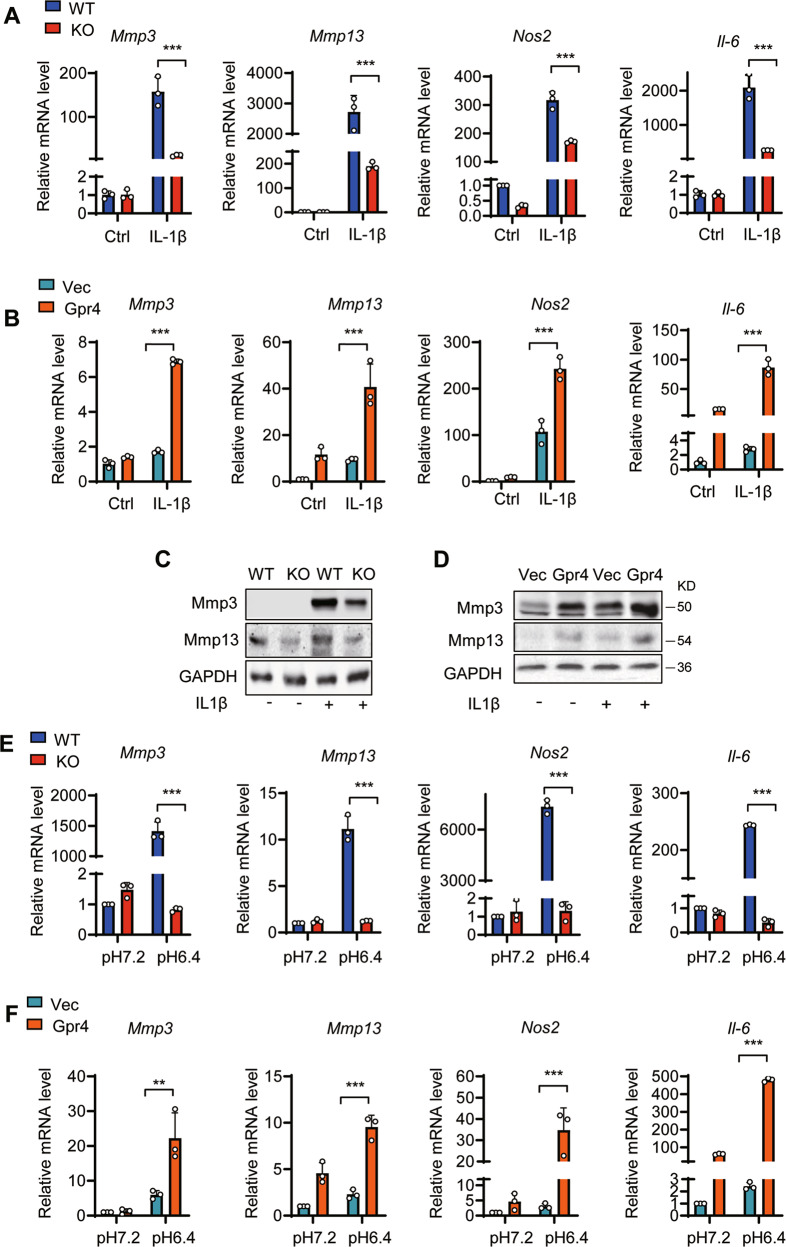
GPR4 regulates cartilage matrix catabolism in mouse chondrocytes. **A** The mRNA levels of *Mmp3*, *Mmp13*, *Nos2*, and *Il-6* were quantified by qRT-PCR in primary chondrocytes from WT and Gpr4 knockout mice. The cells were stimulated with IL-1β (1 ng/ml) for 24 h. *n* = 3. All data are presented as mean ± s.d. ***p < 0.001, using two-way ANOVA followed by Tukey’s post hoc test. **B** The mRNA levels of *Mmp3*, *Mmp13*, *Nos2*, and *Il-6* were quantified by qRT-PCR in ATDC5 cells from vector control (Vec) or Gpr4 transfected cells. Twenty-four hours after transfection, cells were stimulated with IL-1β (1 ng/ml) for another 24 h. *n* = 3. Data are expressed as mean ± s.d. ***p < 0.001, using two-way ANOVA followed by Tukey’s post hoc test. **C**, **D** Western blot analysis of Mmp3 and Mmp13 in chondrocytes of WT and Gpr4 knockout mice (**C**) or vector control (Vec) and Gpr4 transfected ATDC5 cells (**D**). The cells were treated with IL-1β (10 ng/ml) for 48 h prior to harvest. **E**, **F** The mRNA levels of *Mmp3*, *Mmp13*, *Nos2*, and *Il-6* was determined in primary chondrocytes from WT and Gpr4 knockout mice (**E**) or in ATDC5 cells with or without GPR4 transfection (**F**) cultured at the indicated pH value. *n* = 3. Data are expressed as mean ± s.d. **p < 0.01, ***p < 0.001, using two-way ANOVA followed by Tukey’s post hoc test.

To further confirm GPR4 regulation of cartilage destruction in OA, we examined the expression of cartilage anabolic and catabolic marker genes from primary cultured WT and *Gpr4* KO mouse chondrocytes. We found that the expression of the catabolic marker genes *Mmp3*, *Mmp13*, *Nos2*, and *Il-6*, which are important molecules regulating cartilage degradation, was dramatically downregulated in *Gpr4*^*−/−*^ chondrocytes after IL-1β stimulation while *Mmp3* and *Il-6* expression were reduced after TNF-α stimulation (Fig. 6A and Supplementary Fig. 4A). Similarly, the GPR4 antagonist NE decreased IL-1β-induced *Mmp3*, *Mmp13*, and *Il-6* gene expression (Supplementary Fig. 4B). In contrast, overexpression of GPR4 in ATDC5 cells significantly increased the expression of all the factors in response to IL-1β or TNF-α (Fig. 6B and Supplementary Fig. 4C, D). The decreased Mmp3 and Mmp13 protein levels were also confirmed by western blot analysis (Fig. 6C, D).

Because GPR4 is a proton-activated G protein-coupled receptor, activated by slightly acidic pH [19], we next evaluated whether lower pH-induced GPR4 activation affected catabolic marker gene expression. We found that the expression of the four catabolic marker genes was dramatically upregulated when the pH was decreased to 6.4 in WT chondrocytes, while Gpr4 knockout almost completely abolished the increase of the four catabolic marker genes induced by slightly acidic pH (Fig. 6E). Furthermore, overexpression of GPR4 strikingly upregulated catabolic marker gene expression when the pH was decreased to 6.4 (Fig. 6F).

Moreover, we investigated whether GPR4 affects chondrogenesis. We found that knock out of *Gpr4* increased the staining of proteoglycan in primary chondrocytes compared with WT chondrocytes (Supplementary Fig. 5A, B). Consistent with this, *Gpr4* knockout significantly increased the expression of the chondrocyte differentiation markers *Col2a1*, *Acan*, and *Sox9* (Supplementary Fig. 5C). Collectively, all the results indicate that GPR4 played a catabolic function in chondrocytes by promoting cartilage catabolism and suppressing chondrogenic differentiation.

### GPR4 regulates cartilage homeostasis via the CXCL12/CXCR7 signaling pathway

**Fig. 7:**
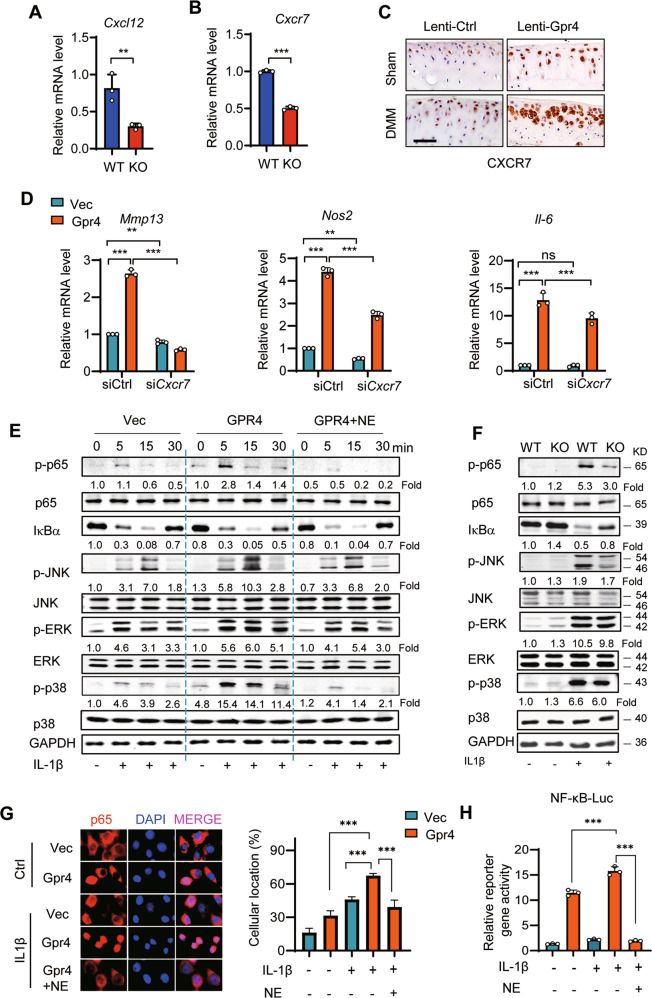
GPR4 regulates cartilage matrix homeostasis through the CXCR7/CXCL12 signaling pathway. **A** The mRNA expression of *Cxcl12* in primary chondrocytes from WT or littermate Gpr4 KO mice. *n* = 3. Data are presented as mean ± s.d. **p < 0.01, using unpaired two-tailed Student’s *t* test. **B** The mRNA expression of *Cxcr7* in primary chondrocytes from WT or littermate Gpr4 KO mice was evaluated. *n* = 3. Data are presented as mean ± s.d. ***p < 0.001, using unpaired two-tailed Student’s *t* test. **C** Representative images of IHC staining of CXCR7 in articular cartilage sections from Sham- or DMM-operated mice with Lenti-Gpr4 or Lenti-Ctrl infection (refer to Fig. 2B). Scale bars, 50 μm. **D** *Cxcr7* knockdown reduced GPR4 overexpression-induced cartilage catabolic gene *Mmp13*, *Nos2*, and *Il-6* upregulation. Vector control (Vec) or Gpr4 overexpressing ATDC5 cells were transfected with the control small interfering RNA (siCtrl) or si*Cxcr7* and exposed to IL-1β (1 ng/ml) for 24 h. Expression of mRNA was analyzed by qRT-PCR. *n* = 3. Data are presented as mean ± s.d. **p < 0.01, ***p < 0.001, ns, no significant difference using two-way ANOVA followed by Tukey’s post hoc test. **E** ATDC5 cells were transfected with vector control or Gpr4, and incubated with NE52-QQ57 (NE, 10 μM) for 24 h. Then the cells were stimulated with IL-1β (10 ng/ml) for indicated times. Whole-cell lysates were subjected to Western blot analysis to determine the total protein and phosphorylation levels of MAPKs, NF-κB (p65), and IκBα. **F** Chondrocytes from GPR4 KO mice and WT mice were stimulated with or without IL-1β (10 ng/ml) for 5 min. The protein and phosphorylation levels of MAPKs, NF-κB (p65), and IκBα were evaluated by Western blot. **G** Representative immunostaining images of p65 nuclear localization (left) and quantification of the percentage of cells with nuclear p65 (right). GPR4 overexpressing ATDC5 cells were treated with NE52-QQ57 (NE, 10 μM) or IL-1β (1 ng/ml) for 12 h, and then subjected to immunofluorescence staining. Data are presented as mean ± s.d. ***p < 0.001, one-way ANOVA followed by Bonferroni’s test was used for statistical analysis. **H** NF-κB reporter gene activity in GPR4 overexpressing ATDC5 cells treated with NE52-QQ57 (NE, 10 μM) or IL-1β (1 ng/ml) for 12 h. Data are presented as mean ± s.d. ***p < 0.001, one-way ANOVA followed by Bonferroni’s test was used for statistical analysis.

To elucidate the possible mechanisms by which GPR4 regulated the pathogenesis of OA, we performed a functional assay to predict the signaling pathway(s) downstream of GPR4 by using 22 OA-related signaling pathway inhibitors (Supplementary Table 1). Our data showed that overexpression of Gpr4 in ATDC5 cells increased Mmp3 expression (~2.5-fold) following IL-1β stimulation (Supplementary Fig. 6A Left). We found that 7 inhibitors downregulated Mmp3 expression (Blue bar) while 15 inhibitors upregulated Mmp3 expression (red bar) (Supplementary Fig. 6A Right). The blue bar indicates that GPR4 positively regulated the signaling pathway this inhibitor affected, while the red bar indicates that GPR4 negatively regulated this signaling pathway. Therefore, we chose the positive signaling pathways for further study. Our data showed that the 3 inhibitors that most decreased MMP3 levels were JSH-23, PD98059, and UNBS5162, which inhibit NF-κB, MEK, and pan-CXCL chemokine expression, respectively (Supplementary Fig. 6A Right). Because CXCL chemokines can activate both NF-κB and MEK signaling for OA progression [37], we next asked whether GPR4 regulated OA progression via the CXCL-NF-κB/MEK signaling pathway. We found that the pan-CXCL chemokine inhibitor UNBS5162 suppressed GPR4-induced upregulation of *Mmp3* expression in a dose-dependent manner (Supplementary Fig. 6B). To find out which CXCL members were regulated by GPR4, we evaluated the effects of these conditions on expression of all Cxcl family members. Our data showed that Gpr4 only upregulated the expression of *Cxcl12* (Supplementary Fig. 6C). Our result of Gpr4 regulating *Cxcl12* was further confirmed in Gpr4 knockout chondrocytes and human OA cartilage (Fig. 7A and Supplementary Fig. 7A). And it is reported that CXCL12 can regulate chondrocyte differentiation and survival and enhance chondrocyte catabolic activity by promoting the release of matrix MMP-3 and MMP-13 [37, 38], suggesting that CXCL12 could be an important factor in the development of OA. As Cxcl12 activates both receptors (Cxcr4 and Cxcr7), we next explored which receptor Cxcl12 activated in OA. Our data showed that only the expression of *Cxcr7*, a higher affinity Cxcl12 receptor, was significantly increased by Gpr4 overexpression in response to either pro-inflammatory cytokines or acidic conditions (Supplementary Fig. 6D). The result was confirmed in *Gpr4* knockout chondrocytes by qRT-PCR (Fig. 7B) and GPR4 overexpressing postromantic OA mouse joints and human articular cartilage tissues by IHC staining (Fig. 7C and Supplementary Fig. 7B). Furthermore, Cxcr7 knockdown decreased the GPR4 induction of *Mmp13*, *Nos2*, and *Il-6* expression under IL-1β stimulation (Fig. 6D and Supplementary Fig. 7C). All the results indicated that GPR4 regulated expression of catabolic markers via Cxcl12 and Cxcr7 signaling.

To further confirm whether GPR4 regulated NF-κB and MEK signaling, we employed western blotting, nuclear translocation staining, and luciferase reporter gene assay. Our results revealed that IL-1β induced phosphorylation of NF-κB (p65), ERK, and JNK, and promoted IκBα degradation in both ATDC5 cells (Fig. 7E) and WT primary chondrocyte cells (Fig. 7F), GPR4 overexpression enhanced NF-κB, ERK, p38, and JNK phosphorylation (Fig. 7E), and *Gpr4* knockout abolished these phosphorylation and degradation changes (Fig. 7F). Furthermore, the GPR4 antagonist NE suppressed GPR4 overexpression-induced NF-κB, ERK, p38, and JNK phosphorylation (Fig. 7E). Consistent results were obtained from the nuclear translocation staining of p65 following IL-1β stimulation, demonstrating that GPR4 overexpression promoted p65 nuclear translocation, while NE treatment suppressed this translocation (Fig. 7G). As a result, GPR4 enhanced NF-κB luciferase reporter gene expression, while the GPR4 antagonist NE strikingly reduced this reporter gene expression (Fig. 7H).

## Discussion

At present, drugs for treating OA are mainly anti-inflammatory and analgesic, without effective therapies for curing OA [39]. The proton-activated GPCRs participate in various physiological functions, including cancer, intestinal inflammation, and angiogenesis. This is the first study, to our knowledge, of the proton-activated G-protein coupled receptor GPR4 in OA pathogenesis. In the present study, we found that GPR4 expression levels were significantly increased in OA patients and in the articular cartilage of OA mouse models. GPR4 deletion prevented the development of posttraumatic and aging-associated OA in mice, while gain-of-function experiments further showed that higher expression levels of GPR4 promoted OA pathogenesis. Mechanistically, GPR4 promoted OA pathogenesis via activating the CXCL12/CXCR7-NF-κB/MEK signaling pathway. Collectively, our results indicate that GPR4 might be a promising therapeutic target for OA.

An acidic environment is a hallmark of the inflammation process. Extracellular acidosis triggered the maturation of human dendritic cells (DCs) and the production of IL-12 [40], and extracellular acidification induced cartilage destruction [41]. Further reports suggested that although the synovial fluid pH value was not changed in OA patients, however, locally inflamed cartilage had become acidic [15–18]. Our data showed that an acidic environment significantly increased the gene expression of catabolic markers and proinflammatory cytokines in chondrocyte cells, and overexpression of GPR4 strikingly upregulated expression of these markers when under slightly acidic conditions, indicating that inhibition of the proton-activated GPCR can prevent cartilage destruction and inflammation.

OA is a whole-joint disease, accompanied by cartilage destruction, synovial inflammation, osteophyte development, and subchondral bone sclerosis [42]. Recent studies found that GPR4 is involved in many inflammatory processes. *Gpr4*^*−/−*^ mice had accelerated clearance of airway inflammation and neutrophils in the cigarette smoke-induced chronic obstructive bowel disease mouse model [43]. Activation of GPR4 in endothelial cells induces the expression of inflammatory genes including *CXCL2*, *CCL20*, *VCAM1*, and *COX2* [23]. The absence of GPR4 ameliorates colitis with lower histology scores, reduced CD4^+^ T helper cell infiltration, and decreased *IFN-γ*, *iNOS*, *CXCL1*, and *CXCL2* expression [44]. Indeed, in both posttraumatic and aging-associated OA models, we found that *Gpr4*^*−/−*^ mice had reduced cartilage degradation and inflammatory infiltration with a decreased percentage of F4/80 positive cells, whereas IA injection of Lenti-Gpr4 promoted synovial inflammation, an effect that was blocked by the GPR4 antagonist NE52-QQ57. Synovial macrophage infiltration plays an important role in triggering OA inflammation. Previous studies showed that GPR4 was expressed in macrophages, and IFN-γ, as a GPR4 downstream signal, induced *iNOS* expression and activated macrophages [45]. These results indicate that GPR4 may play an important role in modulating the synovitis often present in OA. Furthermore, patients with OA experience pain as the most disabling symptom [39]. GPR4 is expressed in dorsal root ganglion (DRG) neurons, which are the primary neurons of pain sensation, while a GPR4 antagonist alleviated inflammatory pain in the rat CFA model [27]. Consistent with these reports, our results showed that deletion and inhibition of GPR4 relieved OA synovitis and pain. Furthermore, osteophyte formation occurs during OA progression. In our study, IA injection of Lenti-Gpr4 or GPR4 antagonists inhibited osteophyte formation. However, there was little difference in osteophyte formation between WT and GPR4 global knockout OA mice, suggesting that subchondral bone hemostasis could have been compensated in GPR4 knockout OA mice during skeleton development. Therefore, targeting GPR4 could be a promising therapy strategy for OA treatment and symptomatic relief, because inhibition of GPR4 locally in joints reduced cartilage catabolism, OA pain, osteophyte formation, promoted cartilage anabolism, and improved joint function.

Previous studies have shown that CXCL12 is secreted by fibroblast-like synoviocytes (FSs), and its receptors, CXCR4, and CXCR7, are both expressed on chondrocytes [38, 46]. CXCL12 induces chondrocyte hypertrophy and modulates multiple homeostatic and pathological processes in the progression of OA [47]. The CXCL12/CXCR7 axis in synovial tissue plays an important role in the development of arthritis [48]. Moreover, mTORC1 activation promotes the secretion of CXCL12 by osteoblasts, leading to abnormal subchondral bone formation and articular cartilage degeneration [49]. Similarly, we found that *Cxcl12* and its receptor *Cxcr7* are downregulated in *Gpr4* KO chondrocytes. We speculate that CXCL12/CXCR7 signaling plays an important role in the regulation of matrix-degrading enzymes induced by GPR4. Consistent with our hypothesis, we found that inhibiting the expression of CXCR7 can reduce the expression of *Il-6*, *Mmp13*, and *Nos2* induced by GPR4. CXCR7 activates multiple signaling pathways including the PI3K/AKT and MAPK pathways [50]. CXCR7 expression influenced phosphorylation of ERK1/2 and p38 but not JNK to regulate hepatocellular carcinoma progression [51]. Our results indicated that the CXCL12/CXCR7 axis triggers activation of MAPK signaling proteins, including ERK1/2 and JNK. In addition, our data showed that the level of phosphorylated p65 was downregulated by GPR4, supported by evidence that CXCL12 axis-activated AKT and ERK signaling pathways have been reported to promote NF-κB nuclear localization, and increase NF-κB signaling by p65 phosphorylation and destabilization of IκBα. Meanwhile, NE52-QQ57, an antagonist of GPR4, successfully inhibited phosphorylation of IκBα and NF-κB activation.

In summary, our findings demonstrated that the proton-activated GPCR GPR4 mediates the pathogenesis of OA by activating the CXCL12/CXCR7 axis, and a GPR4 antagonist effectively ameliorates OA development in both mouse models and human articular cartilage explants. Altogether, the results support that GPR4 could be a promising therapeutic target for OA.

## Supplementary information

Gpr4 OA supplemental materials

Reproducibility checklist

Author Contribution Statement

## Data Availability

The datasets generated in this study are available from the corresponding author on reasonable request.

## References

[1] Roos EM, Arden NK (2016). Strategies for the prevention of knee osteoarthritis. Nat Rev Rheumatol.

[2] Disease GBD, Injury I, Prevalence C. (2018). Global, regional, and national incidence, prevalence, and years lived with disability for 354 diseases and injuries for 195 countries and territories, 1990–2017: a systematic analysis for the Global Burden of Disease Study 2017. Lancet.

[3] Sanchez-Adams J, Leddy HA, McNulty AL, O’Conor CJ, Guilak F (2014). The mechanobiology of articular cartilage: bearing the burden of osteoarthritis. Curr Rheumatol Rep.

[4] Zheng W, Li X, Liu D, Li J, Yang S, Gao Z (2019). Mechanical loading mitigates osteoarthritis symptoms by regulating endoplasmic reticulum stress and autophagy. FASEB J.

[5] Jayakumar T, Saravana Bhavan P, Sheu JR (2020). Molecular targets of natural products for chondroprotection in destructive joint diseases. Int J Mol Sci.

[6] Blom AB, van Lent PL, Libregts S, Holthuysen AE, van der Kraan PM, van Rooijen N (2007). Crucial role of macrophages in matrix metalloproteinase-mediated cartilage destruction during experimental osteoarthritis: involvement of matrix metalloproteinase 3. Arthritis Rheum.

[7] Glasson SS, Askew R, Sheppard B, Carito B, Blanchet T, Ma HL (2005). Deletion of active ADAMTS5 prevents cartilage degradation in a murine model of osteoarthritis. Nature.

[8] Mengshol JA, Vincenti MP, Coon CI, Barchowsky A, Brinckerhoff CE (2000). Interleukin-1 induction of collagenase 3 (matrix metalloproteinase 13) gene expression in chondrocytes requires p38, c-Jun N-terminal kinase, and nuclear factor kappaB: differential regulation of collagenase 1 and collagenase 3. Arthritis Rheum.

[9] Sakao K, Takahashi KA, Arai Y, Saito M, Honjo K, Hiraoka N (2009). Osteoblasts derived from osteophytes produce interleukin-6, interleukin-8, and matrix metalloproteinase-13 in osteoarthritis. J Bone Min Metab.

[10] Wu D, Jin S, Lin Z, Chen R, Pan T, Kang X (2018). Sauchinone inhibits IL-1beta induced catabolism and hypertrophy in mouse chondrocytes to attenuate osteoarthritis via Nrf2/HO-1 and NF-kappaB pathways. Int Immunopharmacol.

[11] Huber V, Camisaschi C, Berzi A, Ferro S, Lugini L, Triulzi T (2017). Cancer acidity: an ultimate frontier of tumor immune escape and a novel target of immunomodulation. Semin Cancer Biol.

[12] Ricciardolo FL, Gaston B, Hunt J (2004). Acid stress in the pathology of asthma. J Allergy Clin Immunol.

[13] Arnett TR (2008). Extracellular pH regulates bone cell function. J Nutr.

[14] Arnett TR (2010). Acidosis, hypoxia and bone. Arch Biochem Biophys.

[15] Geborek P, Saxne T, Pettersson H, Wollheim FA (1989). Synovial fluid acidosis correlates with radiological joint destruction in rheumatoid arthritis knee joints. J Rheumatol.

[16] Ward TT, Steigbigel RT (1978). Acidosis of synovial fluid correlates with synovial fluid leukocytosis. Am J Med.

[17] Jebens EH, Monk-Jones ME (1959). On the viscosity and pH of synovial fluid and the pH of blood. J Bone Jt Surg Br.

[18] Konttinen YT, Mandelin J, Li TF, Salo J, Lassus J, Liljestrom M (2002). Acidic cysteine endoproteinase cathepsin K in the degeneration of the superficial articular hyaline cartilage in osteoarthritis. Arthritis Rheum.

[19] Ludwig MG, Vanek M, Guerini D, Gasser JA, Jones CE, Junker U (2003). Proton-sensing G-protein-coupled receptors. Nature.

[20] Holzer P (2009). Acid-sensitive ion channels and receptors. Handb Exp Pharmacol.

[21] Mahadevan MS, Baird S, Bailly JE, Shutler GG, Sabourin LA, Tsilfidis C (1995). Isolation of a novel G protein-coupled receptor (GPR4) localized to chromosome 19q13.3. Genomics.

[22] An S, Tsai C, Goetzl EJ (1995). Cloning, sequencing and tissue distribution of two related G protein-coupled receptor candidates expressed prominently in human lung tissue. FEBS Lett.

[23] Chen A, Dong L, Leffler NR, Asch AS, Witte ON, Yang LV (2011). Activation of GPR4 by acidosis increases endothelial cell adhesion through the cAMP/Epac pathway. PLoS ONE.

[24] Okito A, Nakahama K, Akiyama M, Ono T, Morita I (2015). Involvement of the G-protein-coupled receptor 4 in RANKL expression by osteoblasts in an acidic environment. Biochem Biophys Res Commun.

[25] Jing Z, Xu H, Chen X, Zhong Q, Huang J, Zhang Y (2016). The proton-sensing G-protein coupled receptor GPR4 promotes angiogenesis in head and neck cancer. PLoS ONE.

[26] Dong L, Krewson EA, Yang LV (2017). Acidosis activates endoplasmic reticulum stress pathways through GPR4 in human vascular endothelial cells. Int J Mol Sci.

[27] Velcicky J, Miltz W, Oberhauser B, Orain D, Vaupel A, Weigand K (2017). Development of selective, orally active GPR4 antagonists with modulatory effects on nociception, inflammation, and angiogenesis. J Med Chem.

[28] Liu H, Liu Y, Chen B (2020). Antagonism of GPR4 with NE 52-QQ57 and the suppression of AGE-induced degradation of type II collagen in human chondrocytes. Chem Res Toxicol.

[29] Wang F, Ma L, Ding Y, He L, Chang M, Shan Y (2021). Fatty acid sensing GPCR (GPR84) signaling safeguards cartilage homeostasis and protects against osteoarthritis. Pharm Res.

[30] Glasson SS, Blanchet TJ, Morris EA (2007). The surgical destabilization of the medial meniscus (DMM) model of osteoarthritis in the 129/SvEv mouse. Osteoarthr Cartil.

[31] Kumar R, Gronhaug KM, Afseth NK, Isaksen V, de Lange Davies C, Drogset JO (2015). Optical investigation of osteoarthritic human cartilage (ICRS grade) by confocal Raman spectroscopy: a pilot study. Anal Bioanal Chem.

[32] Kamekura S, Hoshi K, Shimoaka T, Chung U, Chikuda H, Yamada T (2005). Osteoarthritis development in novel experimental mouse models induced by knee joint instability. Osteoarthr Cartil.

[33] Krenn V, Morawietz L, Burmester GR, Kinne RW, Mueller-Ladner U, Muller B (2006). Synovitis score: discrimination between chronic low-grade and high-grade synovitis. Histopathology.

[34] Little CB, Barai A, Burkhardt D, Smith SM, Fosang AJ, Werb Z (2009). Matrix metalloproteinase 13-deficient mice are resistant to osteoarthritic cartilage erosion but not chondrocyte hypertrophy or osteophyte development. Arthritis Rheum.

[35] Aznan AN, Abdul Karim N, Wan Ngah WZ, Jubri Z (2018). Critical factors for lentivirus-mediated PRDX4 gene transfer in the HepG2 cell line. Oncol Lett.

[36] Rahmati M, Nalesso G, Mobasheri A, Mozafari M (2017). Aging and osteoarthritis: central role of the extracellular matrix. Ageing Res Rev.

[37] Li J, Chen H, Zhang D, Xie J, Zhou X (2021). The role of stromal cell-derived factor 1 on cartilage development and disease. Osteoarthr Cartil.

[38] Kanbe K, Takagishi K, Chen Q (2002). Stimulation of matrix metalloprotease 3 release from human chondrocytes by the interaction of stromal cell-derived factor 1 and CXC chemokine receptor 4. Arthritis Rheum.

[39] Hunter DJ, Bierma-Zeinstra S (2019). Osteoarthritis. Lancet.

[40] Martinez D, Vermeulen M, von Euw E, Sabatte J, Maggini J, Ceballos A (2007). Extracellular acidosis triggers the maturation of human dendritic cells and the production of IL-12. J Immunol.

[41] Sun C, Wang S, Hu W (2018). Acid-sensing ion channel 1a mediates acid-induced inhibition of matrix metabolism of rat articular chondrocytes via the MAPK signaling pathway. Mol Cell Biochem.

[42] Bian Q, Wang YJ, Liu SF, Li YP (2012). Osteoarthritis: genetic factors, animal models, mechanisms, and therapies. Front Biosci (Elite Ed).

[43] Dong L, Li Z, Leffler NR, Asch AS, Chi JT, Yang LV (2013). Acidosis activation of the proton-sensing GPR4 receptor stimulates vascular endothelial cell inflammatory responses revealed by transcriptome analysis. PLoS ONE.

[44] Wang Y, de Valliere C, Imenez Silva PH, Leonardi I, Gruber S, Gerstgrasser A (2018). The proton-activated receptor GPR4 modulates intestinal inflammation. J Crohns Colitis.

[45] Perez-Rodriguez R, Roncero C, Olivan AM, Gonzalez MP, Oset-Gasque MJ (2009). Signaling mechanisms of interferon gamma induced apoptosis in chromaffin cells: involvement of nNOS, iNOS, and NFkappaB. J Neurochem.

[46] Xu Q, Sun XC, Shang XP, Jiang HS (2012). Association of CXCL12 levels in synovial fluid with the radiographic severity of knee osteoarthritis. J Investig Med.

[47] Li J, Chen H, Zhang D, Xie J, Zhou X (2021). The role of stromal cell-derived factor 1 on cartilage development and disease. Osteoarthr Cartil.

[48] Kuang L, Wu J, Su N, Qi H, Chen H, Zhou S (2020). FGFR3 deficiency enhances CXCL12-dependent chemotaxis of macrophages via upregulating CXCR7 and aggravates joint destruction in mice. Ann Rheum Dis.

[49] Lin C, Liu L, Zeng C, Cui ZK, Chen Y, Lai P (2019). Correction to: Activation of mTORC1 in subchondral bone preosteoblasts promotes osteoarthritis by stimulating bone sclerosis and secretion of CXCL12. Bone Res.

[50] Li T, Liu T, Chen X, Li L, Feng M, Zhang Y (2020). Microglia induce the transformation of A1/A2 reactive astrocytes via the CXCR7/PI3K/Akt pathway in chronic post-surgical pain. J Neuroinflammation.

[51] Lin L, Han MM, Wang F, Xu LL, Yu HX, Yang PY (2014). CXCR7 stimulates MAPK signaling to regulate hepatocellular carcinoma progression. Cell Death Dis.

