# Supplementary material for: The proton-activated G protein-coupled receptor GPR4 regulates the development of osteoarthritis via modulating CXCL12/CXCR7 signaling
Source: Cell Death Dis. 2022 Feb 14;13(2):152. doi: 10.1038/s41419-021-04455-4 (PMC8844071; doi:10.1038/s41419-021-04455-4)

Supplementary Materials

The Proton-Activated G Protein-Coupled Receptor GPR4 Regulates the Development of Osteoarthritis via Modulating CXCL12/CXCR7 Signaling

Rong Li[1#], Zijing Guan[1#], Shuyan Bi[1#], Fanhua Wang[1,2], Liang He[1, 2], Xin Niu[1], Yu You[1], Yuwei Liu[1], Yi Ding[1], Stefan Siwko[3], Ning Wang[4], Ziming Zhang[5*], Yunyun Jin[1*], Jian Luo[1,2*]

Supplementary Figure Legends

Supplementary Tables 1-3

Supplementary Figures 1-7

Supplementary Figure Legends

Supplementary Fig. 1 Overexpression of GPR4 in joints aggravated posttraumatic OA pathogenesis in mice.

(A) Schematic illustration of GPR4 overexpression by intra-articular (IA) injection of lentivirus. Four days after DMM surgery, 10-12-week-old C57/BL6J mice were IA injected with lentivirus expressing GPR4 (Lenti-Gpr4) or empty vector control (Lenti-Ctrl) every week. The joints were collected and subjected to H&E and IHC staining 8 weeks after DMM surgery.

(B) Representative images of IHC staining of GPR4 in articular cartilage and meniscus of wild type (WT) mice (10-12 weeks old) which were intra-articular (IA) injected with lentivirus expressing GPR4 (Lenti-Gpr4) or negative control empty lentivirus (Lenti-Ctrl). The mouse joints were injected two times per week. Eight weeks later, the joints were collected and subjected to IHC staining. n=7.

(C and D) Representative three-dimensional micro-CT images of the knee joints in sagittal view (C). The red arrows indicate osteophytes. Osteophyte formation was scored by assessing osteophyte size (D left) and maturity (D right). n = 7 mice per group. All data were presented as mean ± s.d. * $p<0.05$, ** $p<0.01$ using two-way ANOVA followed by Tukey's post hoc test.

Supplementary Fig. 2 Characterization of GPR4 global knockout mice.

(A) Schematic diagram of generation of Gpr4 knockout mice. 176 bp in exon 2 of Gpr4 was deleted using CRISPR/Cas9 technology. Strike-through indicates deleted nucleotides. The underlined nucleotides indicate guide RNA.

(B) Genotyping of GPR4 WT and KO mice, WT: 704bp, KO:528bp.

(C) IHC staining of GPR4 in the articular cartilage sections of 8-week-old WT and littermate GPR4 KO mice.

(D and E) Representative image of Safranin-O staining of the tibial growth plate from WT and littermate GPR4 KO mice (4-month-old) (D) and statistical analysis of the area of the growth plate using Image J software (E). n = 7 mice. Data is expressed as mean ± s.d. ns, no significant. Student's two-tailed *t* test was used for statistical analysis. Scale bars, 50 μm.

Supplementary Fig. 3 Ablation of GPR4 had little effect on osteophyte formation in post-traumatic and aging-associated OA mouse models.

(A) Osteophyte formation was scored by assessing osteophyte size (left) and maturity (right) from WT mice (n = 7 mice) and *Gpr4* KO littermate mice (n = 6 mice). 8 weeks after DMM surgery, the mouse joints were collected and subjected to osteophyte score analysis. Data are expressed as mean ± s.d. Student's two-tailed T test was used for statistical analysis.

(B) Osteophyte formation was scored by assessing osteophyte size (left) and maturity (right) from WT mice (n = 7 mice) and *Gpr4* KO littermate mice (n = 4 mice) at 12 months of age. Data are expressed as mean ± s.d. Student's two-tailed T test was used for statistical analysis.

Supplementary Fig. 4 GPR4 regulated the expression of matrix-degrading enzymes and inflammatory cytokines in chondrocytes.

(A - C) The mRNA levels of *Mmp3*, *Mmp13*, *Nos2* and *Il-6* were quantified by qRT-PCR in primary chondrocytes from WT mice and *Gpr4 KO* mice (A), in ATDC5 cells, overexpressing GPR4 incubated with NE52-QQ57 (NE) for 24 hours (B) and in ATDC5 cells with or without GPR4 overexpression. The cells were stimulated with indicated concentrations of TNF-α for 24 hours (C). n = 3. All data are presented as mean ± s.d. ** $p<0.01$, *** $p<0.001$, ns, no significant difference. Statistical analysis was performed using one-way ANOVA followed by Bonferroni's post hoc test for (B), two-way ANOVA followed by Tukey's post hoc test for (A) and (C).

(D) qRT-PCR analysis showing efficiency of *Gpr4* overexpression in ATDC5 cells.

Supplementary Fig. 5 GPR4 regulates cartilage matrix anabolism in mouse chondrocytes.

(A-C) Mouse primary chondrocytes from WT or littermate Gpr4 KO mice were isolated, then the cells were micromass-cultured in chondrogenic differentiation medium for 3 days. The cells were subjected to Alcian blue staining (A) and quantified by Image J (B). qRT-PCR was used to determine expression of *Col2al*, *Acan* and *Sox9*(C). n = 3. Data are expressed as mean ± s.d. **P < 0.01, *** p<0.001 by unpaired two-tailed Student's T test.

Supplementary Fig. 6 GPR4 regulated the expression of *Cxcl12* and *Cxcr7*.

(A) Screening 22 different signaling pathway inhibitors for downregulation of GPR4-induced Mmp3 expression. ATDC5 cells were transfected with empty vector or Gpr4 expression construct, then stimulated with IL-1β (1 ng/ml) and different inhibitors for 24h. RNA was extracted and the expression level of *Mmp3* was examined by qPCR. The mRNA levels of *Mmp3* in ATDC5 cells from vector control (Vec) or Gpr4 transfected cells（Left） and the ratio of *Mmp3* expression fold change in the Gpr4 overexpression group versus the vector group following exposure to inhibitors (Right) are shown. The data are normalized to Vehicle. Red columns represent upregulated Mmp3 expression with inhibitors versus Vehicle Control under IL-1β stimulation, and blue columns represent downregulated Mmp3 expression with inhibitors versus Vehicle Control under IL-1β stimulation. Data are presented as mean ± s.d. *** p<0.001, two-way ANOVA followed by Tukey's post hoc test was used for statistical analysis.

(B) The mRNA level of *Mmp3* in GPR4 overexpressing ATDC5 cells treated with the indicated concentrations of UNBS5162 for 24 hours in the presence of IL-1β was

determined. n = 3. Data are presented as mean ± s.d. *** $p<0.001$, one-way ANOVA followed by Bonferroni's post hoc test was used for statistical analysis.

(C) The mRNA level of *Cxcl* family expression in ATDC5 cells transfected with vector or GPR4 for 48 hours. n = 6. Data are presented as mean ± s.d. *** $p<0.001$, Student's two-tailed T test was used for statistical analysis.

(D) The mRNA levels of *Cxcr4* and *Cxcr7* in ATDC5 cells transfected with empty vector or Gpr4 expression construct, in the presence of IL-1β (1 ng/ml), TNF-α (10 ng/ml) and cultured at pH 7.2 or pH 6.5 for 12h, are shown. Data are presented as mean ± s.d. ** $p<0.01$, *** $p<0.001$, two-way ANOVA followed by Tukey's post hoc test was used for statistical analysis.

Supplementary Fig. 7 GPR4 regulated the MAPK/NF-κB signaling pathway.

(A) The mRNA levels of *CXCL12* in intact and damaged regions of articular cartilage from OA patients were determined by qRT-PCR. n = 8. Data are expressed as mean ± s.d. **$p < 0.01$ by Student's two-tailed t test.

(B) Representative images of IHC staining of CXCR7 in intact and damaged regions of articular cartilage from human OA patients (left) and CXCR7 positive cells were quantified by IHC (right). Data are expressed as mean ± s.d. ***$p < 0.001$ by Student's two-tailed t test. Scale bar, 50 μm.

(C) qRT-PCR analysis showing Cxcr7 silencing efficiency of siRNA targeting *Cxcr7* in ATDC5 cells. n =3. Data are presented as mean ± s.d. *** $p<0.001$, one-way ANOVA followed by Bonferroni's post hoc test was used for statistical analysis.

Supplementary Tables

Supplementary Table 1: Information of the inhibitors used in this study

Number	Inhibitor Name	Target of Signaling Pathway
1	Vorinostat (SAHA, MK0683)	HDAC
2	YO-01027	Notch; γ-secretase
3	Staurosporine	PKA; PKC
4	3-TYP	SIRT3
5	UNBS5162	CXCR
6	JSH-23	NF-κB
7	PD98059	MEK
8	CCG-63802	RGS Protein
9	Dorsomorphin	AMPK, Autophagy
10	SB-431542	ALK5/TGF-β receptor
11	JK184	Hedgehog
12	Levamlodipine besylate	Calcium Channel
13	Reversine	Aurora Kinase, Autophagy
14	Pazopanib	c-Kit; FGFR; PDGFR; VEGFR
15	GSK2879552	Histone Demethylase
16	U-73122	5-Lipoxygenase; Phospholipase
17	Calcifediol (monohydrate)	vitamin D3
18	3-Methyladenine	PI3k, Autophagy
19	Rapamycin	mTOR, Autophagy
20	CWHM-12	Integrins
21	Olaparib	PARP
22	Uprosertib	AKT

Supplementary Table 2. Characteristics of specimens from patients with OA

NO.	Age/gender	ICRS Grade	Joint	Use
1	72/M	4	Knee (L)	IHC/qPCR
2	76/M	4	Knee (L)	IHC/qPCR
3	64/F	4	Knee (L)	IHC/qPCR
4	67/F	4	Knee (L)	IHC/qPCR
5	66/F	4	Knee (R)	IHC/qPCR
6	63/F	4	Knee (R)	IHC/qPCR
7	68/F	4	Knee (R)	IHC/qPCR
8	71/F	4	Knee (R)	IHC/qPCR
9	55/F	4	Knee (R)	Explants culture
10	71/F	4	Knee (R)	Explants culture
11	54/M	4	Knee (L)	Explants culture
12	74/F	4	Knee (R)	Explants culture
13	52/F	4	Knee (R)	Explants culture
14	59/F	4	Knee (L)	Explants culture

ICRS, International Cartilage Repair Society

Supplementary Table 3. Primer sequences for qPCR

Gene	Origin	Strand	Sequence	Purpose
Gpr4	Human	S<br>AS	5’GGTGAAACTCTTGCCTCGTC3’<br>5’CTCTTCCGAGACCGCTACAAC	qPCR
Col2a1	Human	S<br>AS	5’TGGACGCCATGAAGGTTTTCT3’<br>5’TGGGAGCCAGATTGTCATCTC’	qPCR
Mmp3	Human	S<br>AS	5’GACAAAGGATACAACAGGGACCAAT3’<br>5’TGAGTGAGTGATAGAGTGGGTACAT3’	qPCR
Mmp13	Human	S<br>AS	5’TTGCAGAGCGCTACCTGAGATCAT3’<br>5’TTTGCCAGTCACCTCTAAGCCGAA3’	qPCR
GAPDH	Human	S<br>AS	5’GGAGCGAGATCCCTCCAAAAT3’<br>5’GGCTGTTGTCATACTTCTCATGG3’	qPCR
Gpr4	Mouse	S<br>AS	5’CTCTCTACATCTTCGTCATCGG3’<br>5’CGGTAGCACAGCAACATGAGTG	qPCR
Col2a1	Mouse	S<br>AS	5’ACCTTGGACGCCATGAAAGT3’<br>5’CGGGAGGTCTTCTGTGATCG3’	qPCR
Mmp3	Mouse	S<br>AS	5’TTGACTCAAGGGTGGATGCTGTCT3’<br>5’GCACATGCTGAACAAAGCACTTCC3’	qPCR
Mmp13	Mouse	S<br>AS	5’GTGTGGAGTTATGATGATGT3’<br>5’TGCGATTACTCCAGATACTG3’	qPCR
GAPDH	Mouse	S<br>AS	5’TGGCCTTCCGTGTTCCTAC3’<br>5’GAGTTGCTGTTGAAGTCGCA3’	qPCR

Sox9	Mouse	S<br>AS	5’AGTACCCGCATCTGCACAAC3’<br>5’ACGAAGGGTCTCTTCTCGCT3’	qPCR
Aggrecan	Mouse	S<br>AS	5’CCCAAGCACAGAGGTAAACAG3’<br>5’CTCACATTGCTCCTGGTCTG3’	qPCR
NOS2	Mouse	S<br>AS	5’GTTCTCAGCCCAACAATACAAGA3’<br>5’GTGGACGGGTCGATGTCAC3’	qPCR
IL-6	Mouse	S<br>AS	5’AGCCCACCAAGAACGATAGTC3’<br>5’GCATCAGTCCCAAGAAGGCA3’	qPCR
Gpr4	Mouse	S<br>AS	5’GCTGGGCGTCTACCTGATG3’<br>5’AGGCGATGCTGATATAGATGTTG3’	qPCR
Cxcr1	Mouse	S<br>AS	5’ TCTGGACTAATCCTGAGGGTG3’<br>5’ GCCTGTTGGTTATTGGAACTCTC3’	qPCR
Cxcr2	Mouse	S<br>AS	5’ ATGCCCTCTATTCTGCCAGAT3’<br>5’ GTGCTCCGGTTGTATAAGATGAC3’	qPCR
Cxcr3	Mouse	S<br>AS	5’ TACCTTGAGGTTAGTGAACGTCA3’<br>5’ CGCTCTCGTTTTCCCCATAATC3’	qPCR
Cxcr4	Mouse	S<br>AS	5’ GACTGGCATAGTCGGCAATG3’<br>5’ AGAAGGGGAGTGTGATGACAAA3’	qPCR
Cxcr5	Mouse	S<br>AS	5’ ATGAACTACCCACTAACCCTGG3’<br>5’ TGTAGGGGAATCTCCGTGCT3’	qPCR
Cxcr6	Mouse	S<br>AS	5’ GAGTCAGCTCTGTACGATGGG3’<br>5’ TCCTTGAACTTTAGGAAGCGTTT3’	qPCR

Cxcr7	Mouse	S<br>AS	5’ AGCCTGGCAACTACTCTGACA3’<br>5’ GAAGCACGTTCTTGTTAGGCA3’	qPCR
Cxcl1	Mouse	S<br>AS	5’ CTGGGATTCACCTCAAGAACATC3’<br>5’CAGGGTCAAGGCAAGCCTC3’	qPCR
Cxcl2	Mouse	S<br>AS	5’ CCAACCACCAGGCTACAGG3’<br>5’GCGTCACACTCAAGCTCTG3’	qPCR
Cxcl3	Mouse	S<br>AS	5’CAAGAAGTTTGCCTCAACCCC3’<br>5’GACTTGCCGCTCTTCAGTATCT3’	qPCR
Cxcl5	Mouse	S<br>AS	5’GTTCCATCTCGCCATTCATGC3’<br>5’GCGGCTATGACTGAGGAAGG3’	qPCR
Cxcl9	Mouse	S<br>AS	5’GGAGTTCGAGGAACCCTAGTG3’<br>5’GGGATTTGTAGTGGATCGTGC3’	qPCR
Cxcr10	Mouse	S<br>AS	5’CCAAGTGCTGCCGTCATTTTC3’<br>5’ GGCTCGCAGGGATGATTTCAA3’	qPCR
Cxcl11	Mouse	S<br>AS	5’GGCTTCCTTATGTTCAAACAGGG3’<br>5’ GCCGTTACTCGGGTAAATTACA3’	qPCR
Cxcl12	Mouse	S<br>AS	5’TGCATCAGTGACGGTAAACCA3’<br>5’ TTCTTCAGCCGTGCAACAATC3’	qPCR
Cxcl13	Mouse	S<br>AS	5’GGCCACGGTATTCTGGAAGC3’<br>5’ GGGCGTAACTTGAATCCGATCTA3’	qPCR
Cxcl14	Mouse	S<br>AS	5’GAAGATGGTTATCGTCACCACC3’<br>5’ CGTTCCAGGCATTGTACCACT3’	qPCR

Cxcl15	Mouse	S<br>AS	5’CAAGGCTGGTCCATGCTCC3’<br>5’ TGCTATCACTTCCTTTCTGTTGC3’	qPCR
Cxcl16	Mouse	S<br>AS	5’CCTTGTCTCTTGCGTTCTTCC3’<br>5’ TCCAAAGTACCCTGCGGTATC3’	qPCR
Cxcl17	Mouse	S<br>AS	5’AGGTGGCTCTTGGAAGGTG3’<br>5’ GGTGACATCGTTTGAGAAATTGC3’	qPCR

S, sense; AS, antisense

Li et al., Supplementary Fig. 1

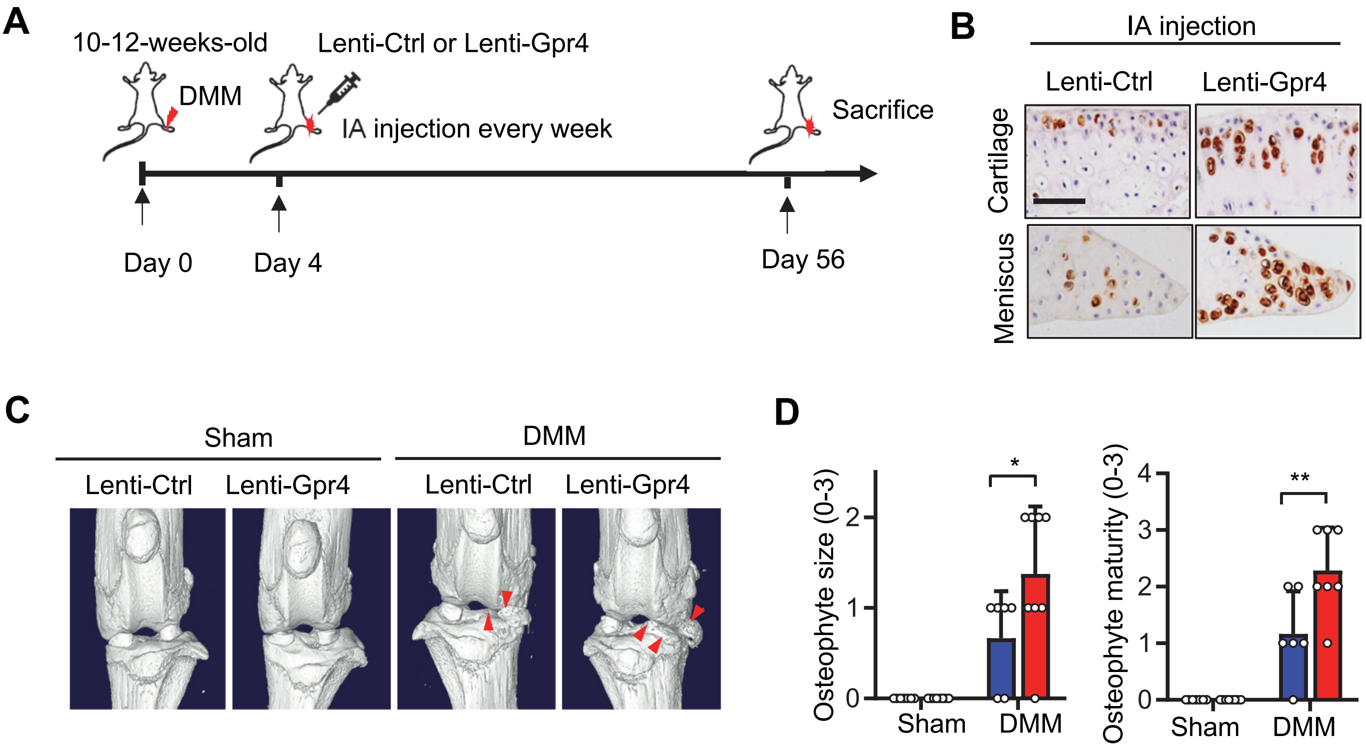

Li et al., Supplementary Fig. 2

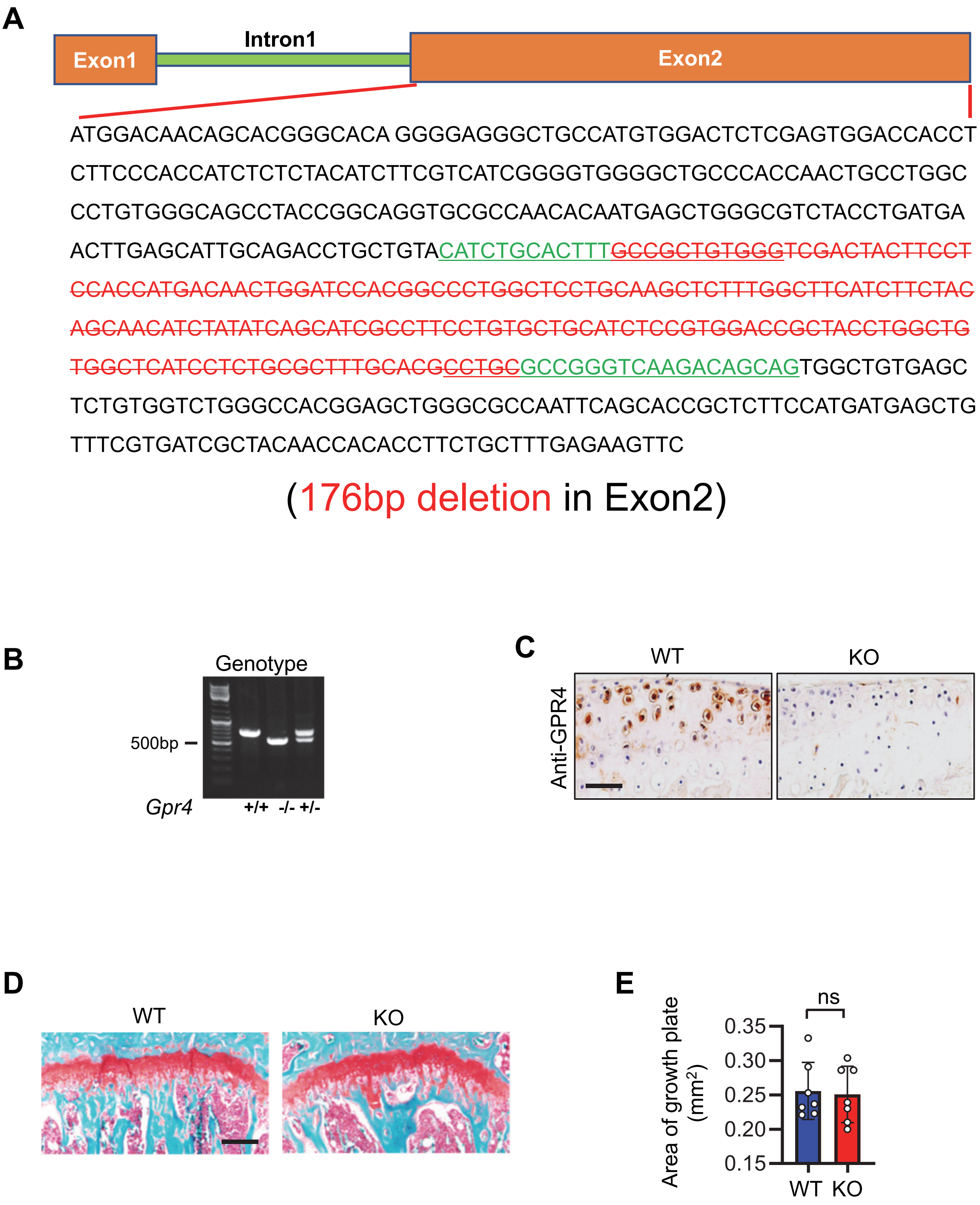

Li et al., Supplementary Fig. 3

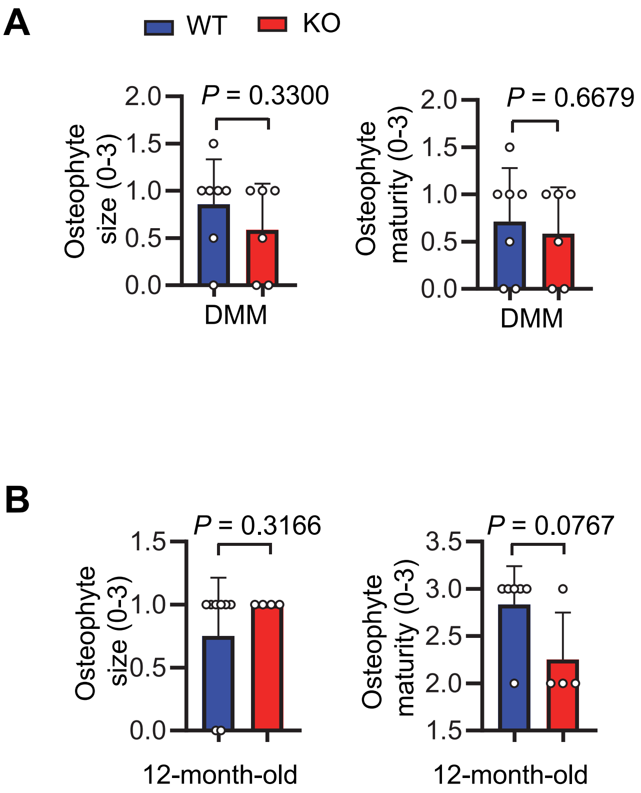

Li et al., Supplementary Fig. 4

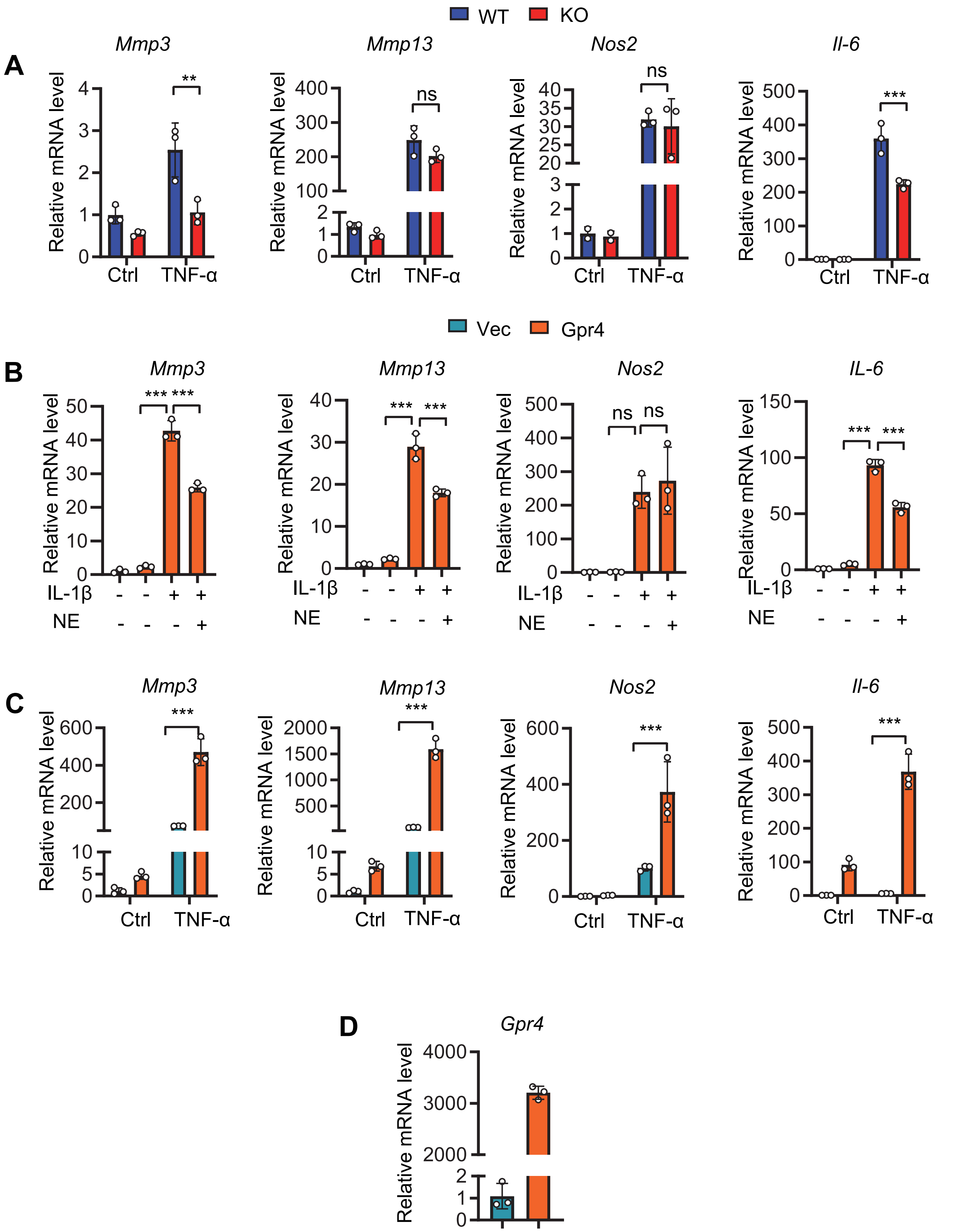

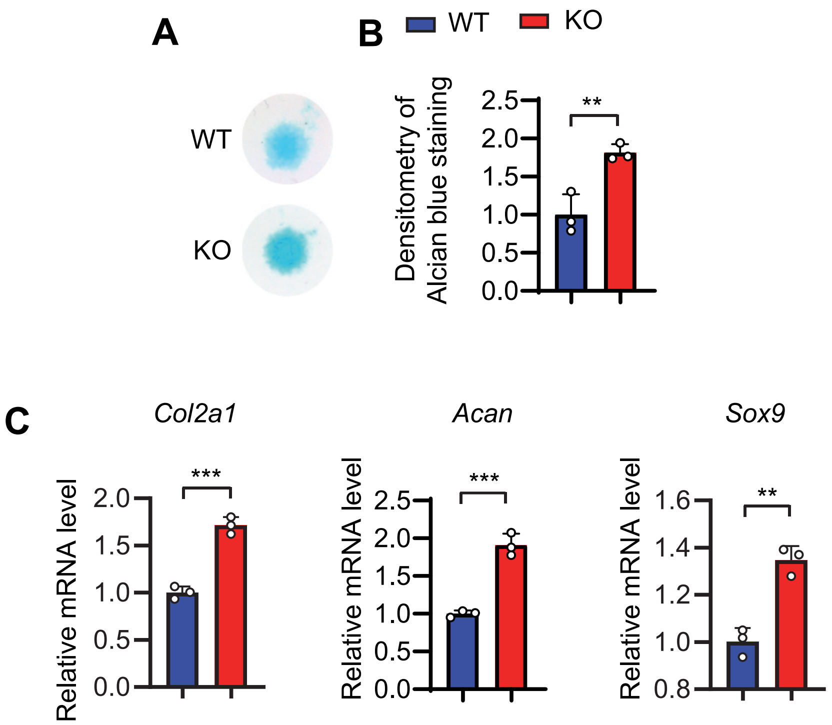
A
WT
KO
B
WT
KO
Densitometry of Alcian blue staining
2.5
2.0
1.5
1.0
0.5
0.0
**
C
Col2a1
Acan
Sox9
Relative mRNA level
***
***
**
2.0
1.5
1.0
0.5
0.0
2.5
1.6
1.4
1.2
1.0
0.8

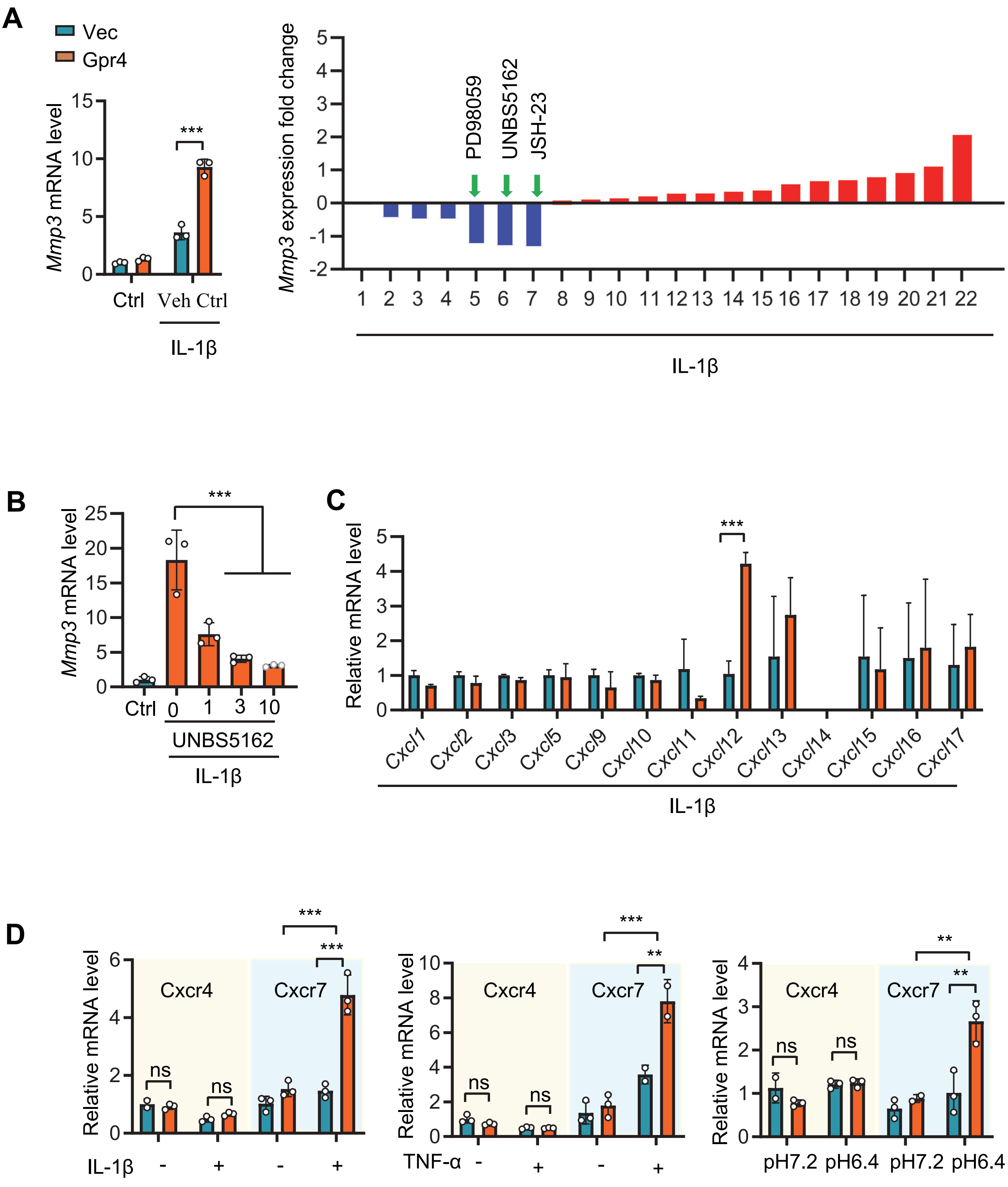
A
Vec
Gpr4
Mmp3 mRNA level
***
Ctrl
Veh Ctrl
IL-1β
Mmp3 expression fold change
PD98059
UNBS5162
JSH-23
1 2 3 4 5 6 7 8 9 10 11 12 13 14 15 16 17 18 19 20 21 22
IL-1β
B
Mmp3 mRNA level
***
Ctrl 0 1 3 10
UNBS5162
IL-1β
C
Relative mRNA level
***
Cxcl1 Cxcl2 Cxcl3 Cxcl5 Cxcl9 Cxcl10 Cxcl11 Cxcl12 Cxcl13 Cxcl14 Cxcl15 Cxcl16 Cxcl17
IL-1β
D
Relative mRNA level
Cxcr4
Cxcr7
ns
***
**
IL-1β - + - +
TNF-α - + - +
pH7.2 pH6.4 pH7.2 pH6.4

Li et al., Supplementary Fig. 7

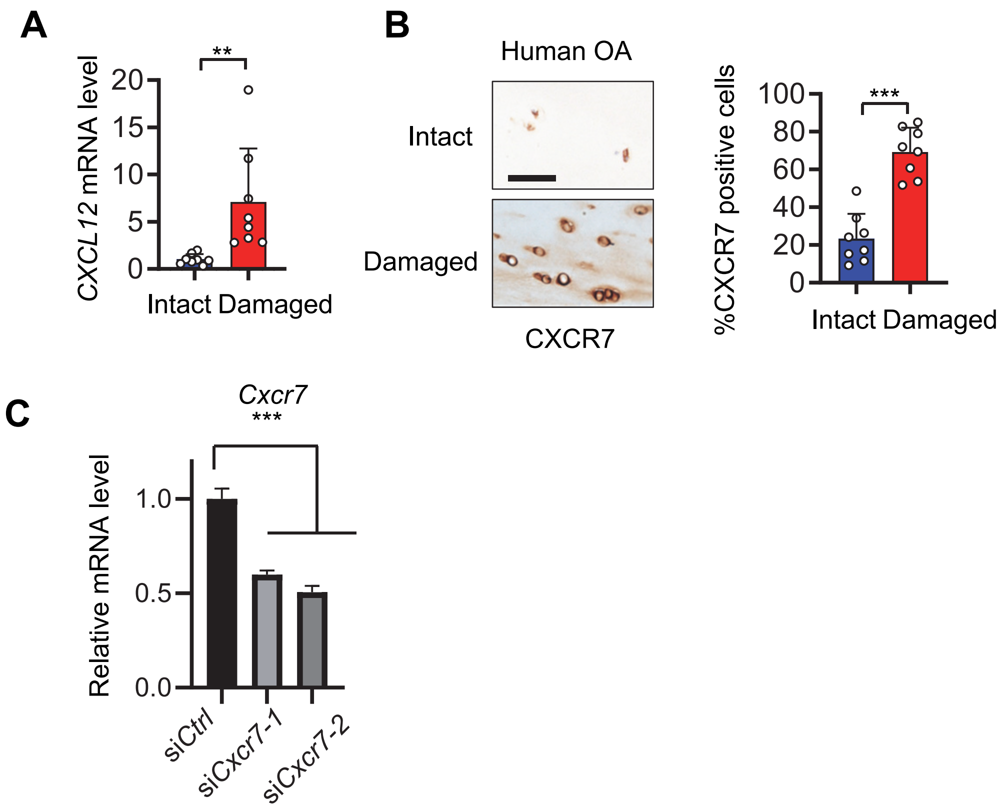

Supplement: Supplementary file 1 — Gpr4 OA supplemental materials [file 41419_2021_4455_MOESM1_ESM.pdf]
