# Supplementary material for: The proton-activated G protein-coupled receptor GPR4 regulates the development of osteoarthritis via modulating CXCL12/CXCR7 signaling
Source: Cell Death Dis. 2022 Feb 14;13(2):152. doi: 10.1038/s41419-021-04455-4 (PMC8844071; doi:10.1038/s41419-021-04455-4)
Supplement: Supplementary file 3 — Author Contribution Statement [file 41419_2021_4455_MOESM3_ESM.docx]

**Authors’ contributions**

JL and RL designed the study and prepared manuscript. ZJG and SYB performed the histological staining and immunohistochemistry. RL and SYB performed the analysis of Western blot, luciferase assay and DMM surgery. FHW and LH collected human cartilage and aging mice cartilage and scored the cartilage section. XN and YY performed primary chondrocyte culture. YD performed micro-CT analysis. YWL analyzed and collected the data. SS, NW and YYJ revised manuscript, ZMZ provided human cartilage. All authors have read and approved the final submitted manuscript.
